# Supplementary material for: A phylogenetically-restricted essential cell cycle progression factor in the human pathogen Candida albicans
Source: Nat Commun. 2022 Jul 23;13:4256. doi: 10.1038/s41467-022-31980-3 (PMC9307598; doi:10.1038/s41467-022-31980-3)
Supplement: Supplementary file 1 — Supplementary Information [file 41467_2022_31980_MOESM1_ESM.pdf]

Supplementary information for

A phylogenetically-restricted essential cell cycle progression factor in the human pathogen *Candida albicans*

Priya Jaitly[1], Mélanie Legrand[2], Abhijit Das[1†], Tejas Patel[1†], Murielle Chauvel[2], Corinne Maufrais[3], Christophe d'Enfert[2*] and Kaustuv Sanyal[1, 4*]

*Corresponding authors. Kaustuv Sanyal (sanyal@jncasr.ac.in); Christophe d'Enfert (christophe.denfert@pasteur.fr)

This PDF file includes:

Supplementary Methods
Supplementary Figures 1 to 13
Supplementary Tables 1 to 5
Supplementary References

Supplementary Methods

Strains, plasmids and primers. Yeast strains, primers and plasmids used in this study are mentioned in supplementary tables 3, 4 and 5, respectively. The *E. coli* library harboring CIp10-P_{TET}-GTW derivatives was constructed in the TOP10 *ccdB*^{R} (Invitrogen) strain of *E. coli* [1]. Other plasmids were propagated in *E. coli* strain DH5α or XL-1 Blue.

Construction of CSA reporter strain

The *RFP* was amplified by PCR using primers RFP-PstI-F and RFP-NheI-R from plasmid pNIM1R-RFP [2]. The PCR fragment was cloned into a TOPO®-TA vector (Thermofisher), digested with PstI and NheI and cloned into the PstI and NheI sites of pTDH3-GFP-URA3 [3], yielding pTDH3-RFP-URA3. The *HYG B* gene was excised from pAU34-CaHygB [4] by BglII+XbaI digest and cloned into the BglII+XbaI double-digested pTDH3-RFP-URA3, to replace the *URA3* marker, yielding pCaTDH3-RFP-HygB. The plasmid pCaTDH3-RFP-HygB was finally modified by a NheI digest and Klenow treatment in order to shorten the extra sequence added at the 3' end because of the cloning steps. The desired P_{TDH3}-*RFP-HygB* cassette was PCR amplified from plasmid pCaTDH3-RFP-HygB with oligonucleotides K7_BFP_GFP_Chr4_Right_F and RFP_Insertion_Chr4_Right_Reverse carrying sequences homologous to the genomic DNA located on the right arm of chromosome 4 (Ch4). The PCR product was then transformed in CEC3867 [5] yielding CEC5201.

Generation of a collection of *C. albicans overexpression strains*

A library of *C. albicans* overexpression strains (1067) was generated using 96-well plates. Briefly, the *E. coli* cultures containing CIp10-P_{TET}-GTW derivatives were grown in 96-deep well plates containing LB or 2YT medium supplemented with ampicillin (50 or 100 µg/ml). Plasmid minipreparations were carried out in 96-well plates using either Nucleospin™ 96 plasmid core kit (Machery-Nagel™) or the boiling lysis method [6]. The quality of the isolated plasmids was randomly checked by ethidium-bromide staining following agarose gel electrophoresis. The plasmids were then digested by either StuI (Anza™ 54 Eco147I) or I-sceI (NEB) depending on

whether a *C. albicans* ORF contained a StuI recognition site [1]. The digested plasmids were precipitated using 3M sodium acetate and 100% ethanol for transformation into the CSA reporter strain (CEC5201), which contains a pNIMX-encoded transactivator to promote the expression from the *TET* promoter [1]. The *C. albicans* transformation was then carried out in 96 deep-well plates using the lithium acetate method, described previously [7]. The *C. albicans* transformants were selected for prototrophy and screened by colony PCR using primers PJ88/PJ89 to confirm the integration of the overexpression plasmid at the *RPS1* locus [1]. The PCR positive transformants were grown in 96 deep-well plates containing YPDU and glycerol stocks of the corresponding strains were prepared.

Construction of C. albicans overexpression strains

StuI-digested or I-*Sce*I-digested CIp10-P_{TET}-GTW derivatives were used to transform *C. albicans* strains in which the pNIMX transactivator cassette was integrated. The integration of pNIMX at the *ADH1* locus of *C. albicans* was carried out after digesting pNIMX with KpnI and ApaI, and confirming the transformants by PCR using primers PJ86/PJ87 [1]. The *C. albicans* transformants harboring the overexpression plasmid at the *RPS1* locus were screened by PCR using primers PJ88 and 89.

C-terminal tagging of Tub4 with fluorescent proteins

GFP-tagged Tub4 expressing strains were constructed by using the plasmid pTub4-GFP-His. Briefly, the 3' coding region of Tub4 without the stop codon was amplified from the *C. albicans* (SN148) genome using primers LS39FP/LS39RP and cloned into the SacII and SpeI sites of pBSGFP-His [8]. The resulting plasmid pTub4-GFP-His was confirmed using restriction analyses, digested with PacI and was used to transform the *C. albicans* strains. The correct *C. albicans* transformants were screened by fluorescence microscopy.

Epitope tagging of Tub4 with mCherry was carried out using the plasmids pTub4-mCherry-Arg4 or pTub4-mCherry-Nat. To construct pTub4-mCherry-Arg4, the C-terminus of Tub4 was released from pTub4-GFP-His following digestion with SacII and SpeI and subsequently cloned

into the SacII and SpeI sites of pRFP-Arg4 [9]. The *E. coli* clones were confirmed by restriction analyses. The plasmid pTub4-mCherry-Arg4 was partially digested with PacI for transforming *C. albicans* strains. The transformants obtained were selected for prototrophy and screened by fluorescence microscopy. The plasmid pTub4-mCherry-Nat was constructed as follows: the mCherry coding gene was amplified from CaADH1pyEmRFP [10] using the primers SR149/SR150 and cloned into the SpeI and SmaI sites of pBSNAT [11]. The mCherry-NAT containing plasmid was then digested by SpeI and KpnI and the mCherry-NAT fragment was cloned into the SpeI and KpnI sites of pTub4-GFP-His. The resulting plasmid pTub4-mCherry-Nat was verified by restriction analyses and used to transform *C. albicans* strains after PacI digestion. The correct *C. albicans* transformants were screened by fluorescence microscopy.

C-terminal tagging of Tub1 with mCherry

The 3' coding region of Tub1 without the stop codon was amplified from *C. albicans* (SN148) genome using the primer pairs PJ77/PJ88 and cloned into the SacII and SpeI sites of pRFP-Arg4 [9]. The resulting plasmid pTub1-mCherry-Arg4 was confirmed using restriction analyses and digested with XbaI for transforming the *C. albicans* strains. The correct *C. albicans* transformants were screened by fluorescence microscopy.

C-terminal tagging of Cse4 with TAP

The *CSE4* ORF (without the stop codon) along with the *TAP* tag was PCR amplified from the *C. albicans* strain CAKS102 [12] using the primers NV241/NV242 and cloned into the SalI and ApaI sites of pMad2-2 [13]. The desired plasmid pCse4-TAP-Leu was verified by restriction analyses and linearized by XhoI for transforming the *C. albicans* strains. The transformants were selected for prototrophy and confirmed by western blot analysis.

Construction of bub2 null mutant

Both the alleles of *BUB2* were deleted using the *SAT1* flipper cassette (pSFS2a) [14]. To delete the first allele, upstream (US) and downstream (DS) sequences of *BUB2* were amplified from the *C.*

albicans (SN148) genome using primers PJ110/PJ111 and PJ112/PJ113, respectively. The US and DS sequences were then cloned in pSFS2a as KpnI/XhoI and SacII/SacI fragments, respectively, to obtain the plasmid pBub2del#1. The *E. coli* clones were verified using restriction analysis. The desired deletion cassette was transformed into the *C. albicans* strains after digesting pBub2del#1 with KpnI and SacI. The *C. albicans* transformants were screened for correct chromosomal integration by PCR using the primer pair PJ3/PJ116. The correct transformants were grown in YPM (1% yeast extract, 2% peptone, 2% maltose) medium supplemented with uridine (0.1µg/ml) overnight and plated on YPMU agar to recycle the *SAT1* marker [14]. The single colonies obtained on YPMU agar were replica plated on YPDU and YPDU with nourseothricin (100 µg/ml). Nourseothricin-sensitive colonies, obtained because of *SAT1* recycling, were reconfirmed for *SAT1* eviction and *BUB2* first copy deletion by PCR using primers PJ110/PJ113 and selected for subsequent transformation experiments to delete the remaining *BUB2* allele.

To delete the second allele of *BUB2*, the DS sequence of *BUB2* in pBub2del#1 was replaced with the 3' coding region of *BUB2*. For this, the 3' coding region of *BUB2* was amplified from the *C. albicans* (SN148) genome using primers PJ114/PJ115 and cloned into the SacII and SacI sites of pBub2del#1. The resulting plasmid pBub2del#2 was verified using restriction analyses and was used to transform the *BUB2* heterozygous null strain after digesting pBub2del#2 with KpnI and SacI. The transformants obtained were grown in presence of nourseothricin (100 µg/ml) and screened for the integration of pBub2del#2 deletion cassette by PCR using the primers PJ117/PJ118. The desired PCR positive transformants were reverified for *BUB2* first copy deletion using the primers PJ110/PJ113. The resulting nourseothricin-resistant *bub2* mutants were grown in YPMU overnight and plated on YPMU agar to recycle the *SAT1* marker [14]. The single colonies obtained on YPMU agar were replica plated on YPDU and YPDU with nourseothricin (100 µg/ml). Nourseothricin-sensitive colonies were selected for subsequent experiments.

Construction of csa6 conditional mutant

The first allele of *CSA6 (ORF19.1447)* was deleted using the *SAT1* flipper cassette (pSFS2a) [14] and the second allele was placed under the control of regulatable *MET3* promoter [15]. To delete the first allele, the US and DS sequences of *CSA6* were amplified from the *C. albicans* (SN148) genome using primers PJ95/PJ96 and PJ97/PJ98, respectively. The US and DS sequences were then cloned in pSFS2a as KpnI/XhoI and SacII/SacI fragments, respectively, to obtain the plasmid pCsa6del. The *E. coli* clones were confirmed using restriction analysis. The desired deletion cassette was transformed into *C. albicans* strains after digesting pCsa6del with KpnI and SacI. The *C. albicans* transformants were screened for correct chromosomal integration by PCR using the primer pair PJ3/PJ99. The correct transformants were grown in YPMU overnight and plated on YPMU agar to recycle the *SAT1* marker [14]. Nourseothricin-sensitive colonies, obtained because of *SAT1* recycling, were selected for subsequent transformations to inactivate the remaining wild-type allele of *CSA6*.

To replace the promoter of the second allele with the *MET3* promoter [15], the 5' coding region of *CSA6* including the start codon was amplified from the *C. albicans* SN148 genome using primers PJ93/PJ94 and cloned into the BamHI and PstI sites of pCaDis [15], generating the plasmid pCsa6-Met3-Ura. The *E. coli* clones were confirmed using restriction analyses. The plasmid pCsa6-Met3-Ura was linearized using BstBI and was used to transform *C. albicans* strains in which the first copy of *CSA6* was deleted. The resulting conditional mutants were screened for correct genomic integration by PCR using the primer pair PJ91/PJ95.

To generate the plasmid pCsa6-Met3-His, a *HIS1* fragment from pGFP-HIS [8] was obtained after digesting pGFP-HIS with EcoRI and was cloned into the EcoRI site of p1447-Met3-His. The *E. coli* transformants were screened and desired clones were validated by restriction analysis.

Epitope tagging of Csa6

The C-terminus of *CSA6* was tagged with either TAP or mCherry. To express TAP-tagged Csa6 from the native promoter or the *MET3* promoter, the 3' coding region of *CSA6* without the stop codon was amplified from the *C. albicans* SN148 genome using the primer pair PJ108 (containing the BglII restriction site) and PJ109 and cloned into the BamHI and PacI sites of

pFA-TAP-*ARG4* [16]. The *E. coli* clones were confirmed by both restriction analyses and Sanger sequencing. The resulting plasmid p1447-TAP-Arg was linearized using BamHI for single-site integration into the *C. albicans* genome. The *C. albicans* transformants were screened for correct genomic integration by PCR using the primer set NV34/TEJ13 and western blot analysis.

To express Csa6TAP from the P_{TET} promoter, a fragment containing the coding region of *CSA6* along with the *TAP* tag was amplified from CaPJ180 using primers PJ127/PJ128 and cloned into the EcoRV site of pCIp10-P_{TET}-GTW [1]. The resulting plasmid CIp10-P_{TET}-1447TAP was confirmed using restriction analyses and Sanger sequencing and was digested by StuI for transforming the *C. albicans* strains. The correct *C. albicans* transformants were screened by PCR using primers PJ88/PJ89 and western blot analyses.

To tag the C-terminus of Csa6 with mCherry, the 3' coding region of *CSA6* without the stop codon was amplified from the *C. albicans* SN148 genome using the primer pair TEJ1/TEJ2 and cloned into the SacII and SpeI sites of pRFP-Arg4 [9]. The *E. coli* clones were confirmed by both restriction analyses and Sanger sequencing. The resulting plasmid pCsa6-mCherry-Arg was linearized using BstB1 for single-site integration into the *C. albicans* genome. The correct *C. albicans* transformants were confirmed by PCR using primers TEJ13/TEJ14 and analyzed by fluorescence microscopy. The functionality of the mCherry-tagged Csa6 was determined by tagging the only copy of *CSA6* in a heterozygous null mutant (CaPJ209, *csa6/CSA6*) with mCherry. The resulting strain CaPJ117 (*csa6/CSA6-mCherry*) was viable and did not show any growth defect.

Construction of SOL1 overexpression mutant

To overexpress Sol1, an extra copy of *SOL1* under the P_{TET} promoter was integrated at the *RPS1* locus [1]. For this, the complete ORF sequence of *SOL1* including the start and the stop codon was PCR amplified from the *C. albicans* (SN148) genome using primers PJ119/PJ120 and cloned into the EcoRV site of CIp10-P_{TET}-GTW [1] resulting in plasmid CIp10-P_{TET}-SOL1. The correct *E. coli* clones were screened using restriction analyses and verified by Sanger sequencing. The

plasmid CIp10-P_{TET}-SOL1 can be linearized by StuI for *C. albicans* transformation. The *C. albicans* transformants were confirmed by PCR using primers PJ88/PJ89.

Epitope tagging of Sol1

The expression level of Sol1 from the native promoter and P_{TET} promoter was compared by tagging the C-terminus of *SOL1* with TAP. For TAP tagging Sol1 under its own promoter, the 3' coding region of *SOL1* without the stop codon was amplified from the *C. albicans* (SN148) genome using the primer pair PJ141/PJ142 and cloned into the BamHI and PacI sites of pFA-TAP-*His1* [15]. The *E. coli* clones were confirmed by both restriction analyses and Sanger sequencing. The resulting plasmid pSol1-TAP-His was linearized using XbaI for single-site integration into the *C. albicans* genome. The *C. albicans* transformants were screened for correct genomic integration by PCR using the primer set PJ119/PJ128 and western blot analysis.

To express Sol1TAP from P_{TET}, a fragment containing the coding region of *SOL1* along with the *TAP* tag was amplified from CaPJ216 using primers PJ119/PJ128 and cloned into the EcoRV site of CIp10-P_{TET}-GTW [1]. The resulting plasmid CIp10-PTET-SOL1TAP was confirmed using restriction analyses and Sanger sequencing. The plasmid CIp10-PTET-SOL1TAP was linearized by StuI for transforming the *C. albicans* strains and the transformants were screened by PCR using primers PJ88/PJ89 and western blot analyses.

Construction of GFP-tagged strains of Tem1, *Spc110* and *Cmd1*

Tem1 and Spc110 were tagged C-terminally with GFP. For this, 3' coding region of Tem1 or Spc110 (orf 19.3100) without the stop codon was amplified from the *C. albicans* (SN148) genome using the primer pairs PJ121/122 and PJ106/PJ107, respectively and cloned into the SacII and SpeI sites of pBSGFP-His [8]. The resulting plasmids (i) pTEM1-GFP-His was confirmed by both restriction analyses and Sanger sequencing (ii) pSpc110-GFP-His was validated by restriction analyses.

The plasmid pTEM1-GFP-His was propagated in the *dam⁻/dcm⁻* strain of *E. coli* (C2925) obtained from NEB and digested with BclI for transforming the *C. albicans* strains. The correct *C. albicans* transformants were screened by PCR using primers PJ123/PJ124 and fluorescence microscopy. The plasmid pSpc110-GFP-His was linearized with NheI or NsiI for single-site integration into the *C. albicans* genome. The *C. albicans* transformants were screened by fluorescence microscopy.

The C-terminus of Cmd1 was tagged with GFP. For this, the 3' coding region of Cmd1 (orf 19.4413) without the stop codon was PCR amplified from the *C. albicans* (SN148) genome using the primer pair PJ157/PJ158 and cloned into the SacII and SpeI sites of pBSGFP-His [8]. The resulting plasmid pCmd1-GFP-His #1 was verified by both restriction analyses and Sanger sequencing. The DS sequence of Cmd1 was then amplified from the *C. albicans* (SN148) genome using the primers PJ159/PJ160 and cloned into the KpnI and HindIII sites of pCmd1-GFP-His #1. The resulting plasmid pCmd1-GFP-His #2 was digested with Kpn1 and SacII for transforming *C. albicans* strains. The correct *C. albicans* transformants were screened by fluorescence microscopy.

C-terminal tagging of Clb2 with TAP

The *CLB2* ORF (without the stop codon) was PCR amplified from the *C. albicans* (SN148) genome using the primers PJ147/PJ148 and cloned into the BamHI and PacI sites of pFA-TAP-*HIS1* [16]. The *E. coli* clones were confirmed by both restriction analyses and Sanger sequencing. The plasmid pClb2-TAP-His was digested with XbaI for transforming *C. albicans* strains. The correct *C. albicans* transformants were screened by western blot analysis.

Expression of C. dubliniensis Csa6 in C. albicans

The C-terminus of *C. dubliniensis* Csa6 (Cd36_16290) was tagged with GFP and ectopically expressed in *C. albicans* using the plasmid pCdCsa6-GFP-ARS2. For this, the complete ORF of *CdCSA6* (without the stop codon), along with its promoter region was PCR amplified from the genome of Cd36, a *C. dubliniensis* clinical isolate [11], using the primers VS5/VS6. The *GFP* tag

was amplified from pTub4-GFP-His using the primers VS7/VS8. An overlap PCR of the two fragments was then set up using the primers VS5/VS8. The resulting ~3.6 kb long fragment containing the GFP-tagged *C. dubliniensis CSA6* under its own promoter was cloned into the XbaI and PstI sites of pARS2 [8]. The plasmid, pCdCsa6-GFP-ARS2, obtained was verified by restriction analyses and transformed into the *C. albicans* strains. The transformants were selected for prototrophy and screened by fluorescence microscopy. As ARS plasmids are highly unstable in *C. albicans*, we used the large transformant colonies, obtained as a result of an integrative transformation of pARS2 [17] and retained the auxotrophic marker (*URA3)* even in the absence of any selection pressure, for all our assays.

Expression of CaCsa6 orthologs of C. tropicalis and C. parapsilosis in C. albicans

Ectopic expression of both *C. tropicalis* Csa6 and *C. parapsilosis* Csa6 was carried out using CIp10 plasmid[18]. For this, the GFP coding gene was PCR amplified from pBSGFP-His [8] using primer pair TEJ15/TEJ18 and cloned into the XhoI and HindIII sites of CIp10. The resulting plasmid pCIp10-GFP was verified by restriction analyses and used for cloning CtCsa6 or CpCsa6. For this, the complete ORF of CtCsa6 (CTRG_01235) or CpCsa6 (CPAR2_214050) without the stop codon, along with its promoter region was PCR amplified from *C. tropicalis* (clinical isolate MYA-3404) or *C. parapsilosis* (NRRL Y-8312) genome using the primers HA004/HA005 and DB2/DB3, respectively and cloned into the Kpn1 and XhoI sites of pCIP10-GFP. The resulting plasmids pCIp10-GFP-CtCsa6 and pCIp10-GFP-CpCsa6 were digested with StuI for transforming *C. albicans*. The *C. albicans* transformants were selected for prototrophy and screened by colony PCR using primers PJ88/PJ89 to confirm the integration of the overexpression plasmid at the *RPS1* locus.

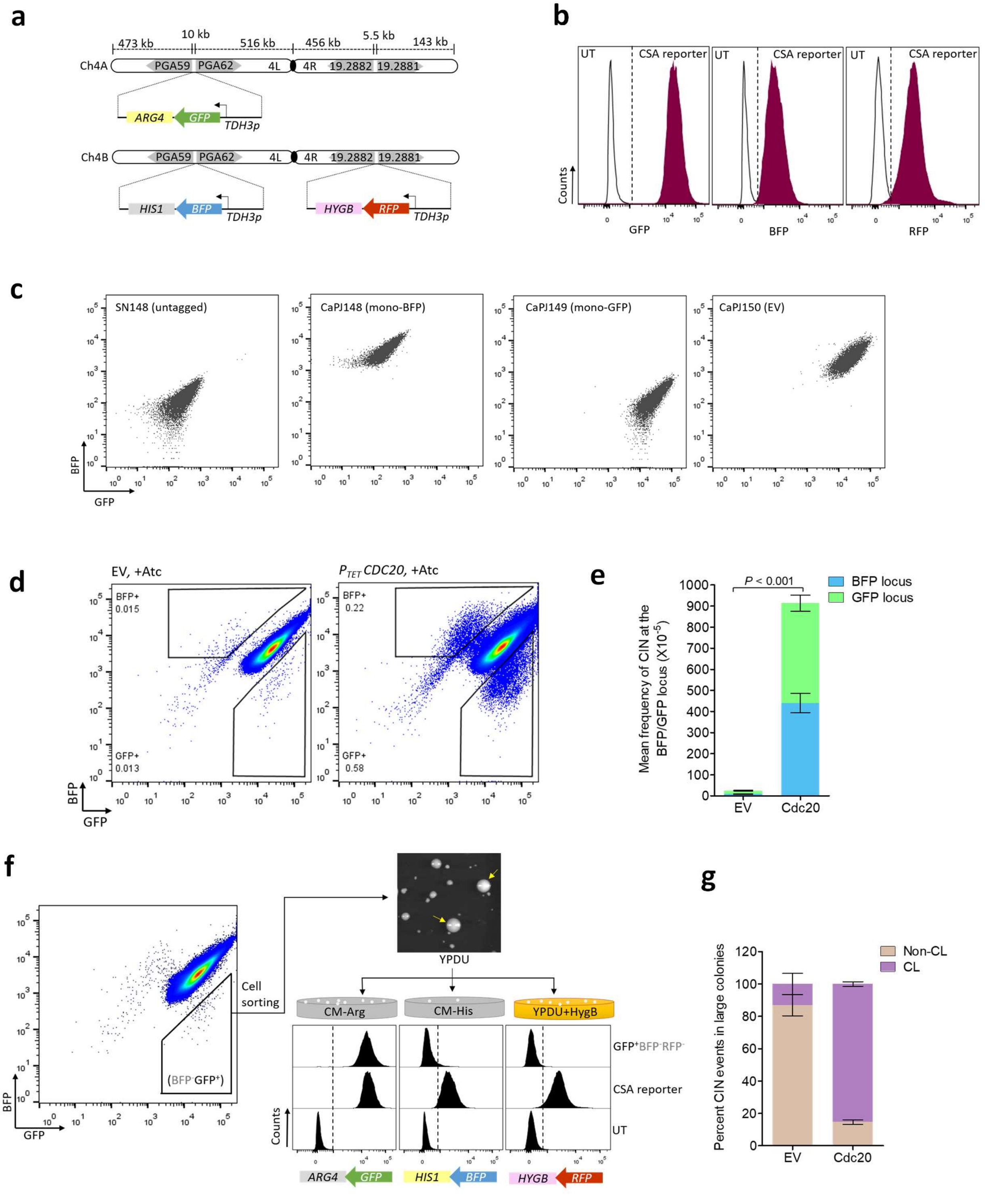

Supplementary Fig. 1. The CSA reporter system for detecting chromosome instability (CIN) in *C. albicans*. (a) A line diagram of Chromosome 4 (Ch4) in the CSA reporter

(CEC5201). As indicated, BFP/GFP-expressing cassettes are present on the left arm of both the homologs of Ch4 in the *PGA59-PGA62* intergenic region, while the RFP-expressing cassette is present on the right arm of Ch4 in the *ORF19.2882-ORF 19.2881* intergenic region. Expression of BFP, GFP or RFP is under control of the *TDH3* promoter and is associated with respective selectable markers, as mentioned in the diagram. **(b)** Histograms showing fluorescence intensity measurements of an untagged strain (SN148) and the CSA reporter strain (CEC5201) by flow cytometry. The CSA reporter strain on the *right* exhibits higher fluorescence intensity for GFP, BFP and RFP laser than the untagged (UT) strain. **(c)** Flow cytometric analysis of the BFP/GFP marker in various control strains as indicated. The strains were grown in YPDU medium overnight and analyzed by flow cytometry. Approximately 10,000 events are displayed. **(d)** Detection of chromosome instability in the *CDC20*^{OE} strain (CaPJ151). *Left*, a representative BFP/GFP density plot of EV in presence of anhydrotetracycline (Atc) (3 μg/ml), an inducer of P_{TET}. The proportion of BFP^{+}GFP^{-} and BFP^{-}GFP^{+} cells in the EV indicates the intrinsic instability of Ch4 in *C. albicans*. *Right*, a representative BFP/GFP density plot of the *CDC20*^{OE} strain in presence of Atc (3 μg/ml). **(e)** Quantitation of the mean frequency (x10^{-5}) of CIN at the BFP/GFP locus in CaPJ150 (EV) and CaPJ151 (*CDC20*^{OE}); *N*=3; mean ± SEM of three independent experiments is indicated. Unpaired *t*-test, one-tailed, *P*-value shows a significant difference (*P*=0.0004 and *P*=0.0001 for BFP and GFP locus, respectively). **(f)** Schematic illustrating the workflow to differentiate CL from non-CL events. A representative flow cytometry density plot is shown as a reference. BFP^{-}GFP^{+} cells were sorted and plated on YPDU agar. Large colonies (arrow marked in yellow) were tested for the presence of selectable markers, *ARG4*, *HIS1* and *HYG B* by replica plating, followed by flow cytometry analysis to monitor the presence of the associated fluorescent proteins (GFP, BFP and RFP). The fluorescence intensity profile of an *ARG4* resistant colony which had lost *HIS1* and *HYG B* (GFP^{+} BFP^{-}RFP^{-}) is shown as an example. The concomitant loss of *BFP-HIS1* and *RFP-HYG B* indicates that the entire Ch4B is lost. **(g)** Analysis of the marker genes, *ARG4*, *HIS1 and HYG B* by replica plating BFP^{-}GFP^{+} colonies of EV (CaPJ150) and *CDC20*^{OE} strain (CaPJ151); *n* ≥299 colonies; mean ± SEM of indicated number of colonies analyzed for each genotype from at least three independent experiments. Source data are provided as a Source Data file.

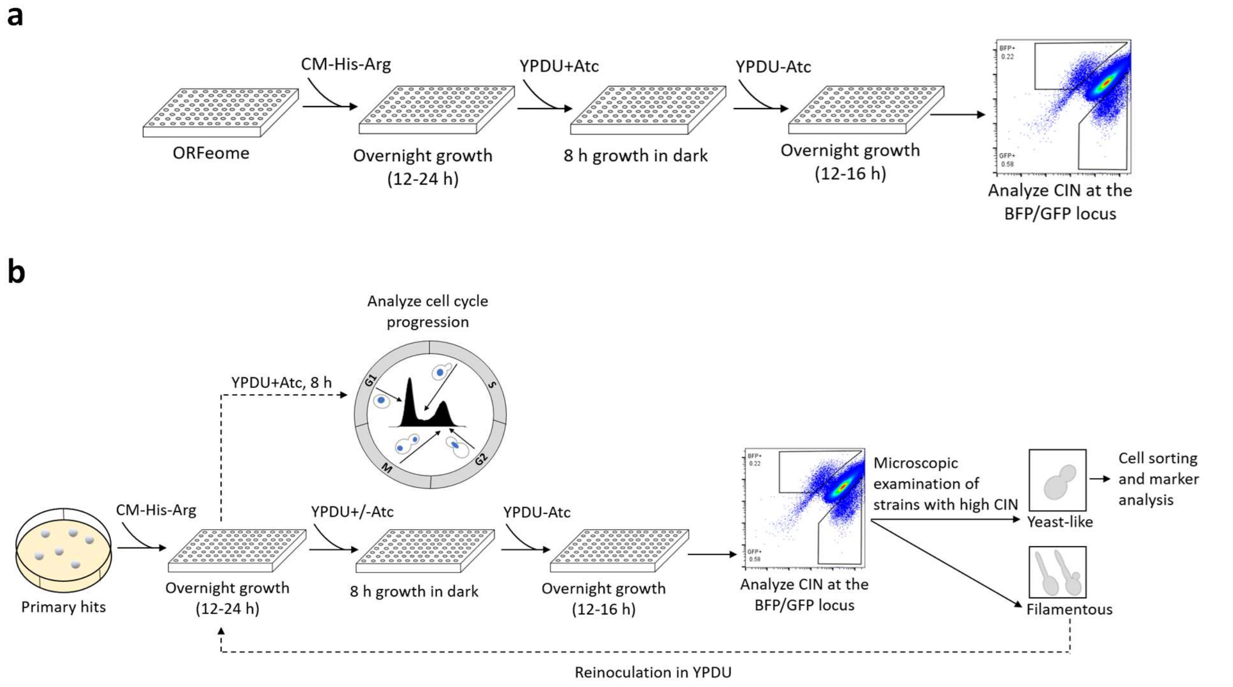

Supplementary Fig. 2. Primary and secondary screening of the *C. albicans* overexpression library. (a) Flow chart illustrating the steps of the primary screen. Briefly, overnight grown cells were induced for 8 h in presence of Atc (3 µg/ml), allowed to recover overnight in a rich medium without Atc, diluted in 1x PBS, and analyzed for BFP/GFP marker by flow cytometry (~10^6 cells). Gates were defined for the BFP^+GFP^- and BFP^-GFP^+ populations in the EV (CaPJ150) and applied to all other over-expression mutants (1067). Mutants were selected if the frequency of CIN at the BFP/GFP locus was two-fold higher than the frequency in the EV. **(b)** Flow diagram illustrating the steps of the secondary screen. The overexpression mutants identified from the primary screen (23 out of 1067) were induced for 8 h in presence or absence of Atc (3 µg/ml), allowed to recover overnight in a rich medium without Atc and analyzed for the loss of BFP and GFP by flow cytometry. Mutants were selected if they exhibited two-fold higher rate of CIN at the BFP/GFP locus in three biological replicates as compared to the EV and further analyzed for any morphological transition by microscopy. Overexpression mutants with yeast-like morphology were analyzed by cell sorting and marker analysis to determine the molecular mechanism (CL or non-CL) leading to CIN. Overexpression mutants exhibiting polarized growth were regrown, induced for 8 h in presence of Atc (3 µg/ml) and analyzed for cell cycle progression by microscopy or flow cytometry.

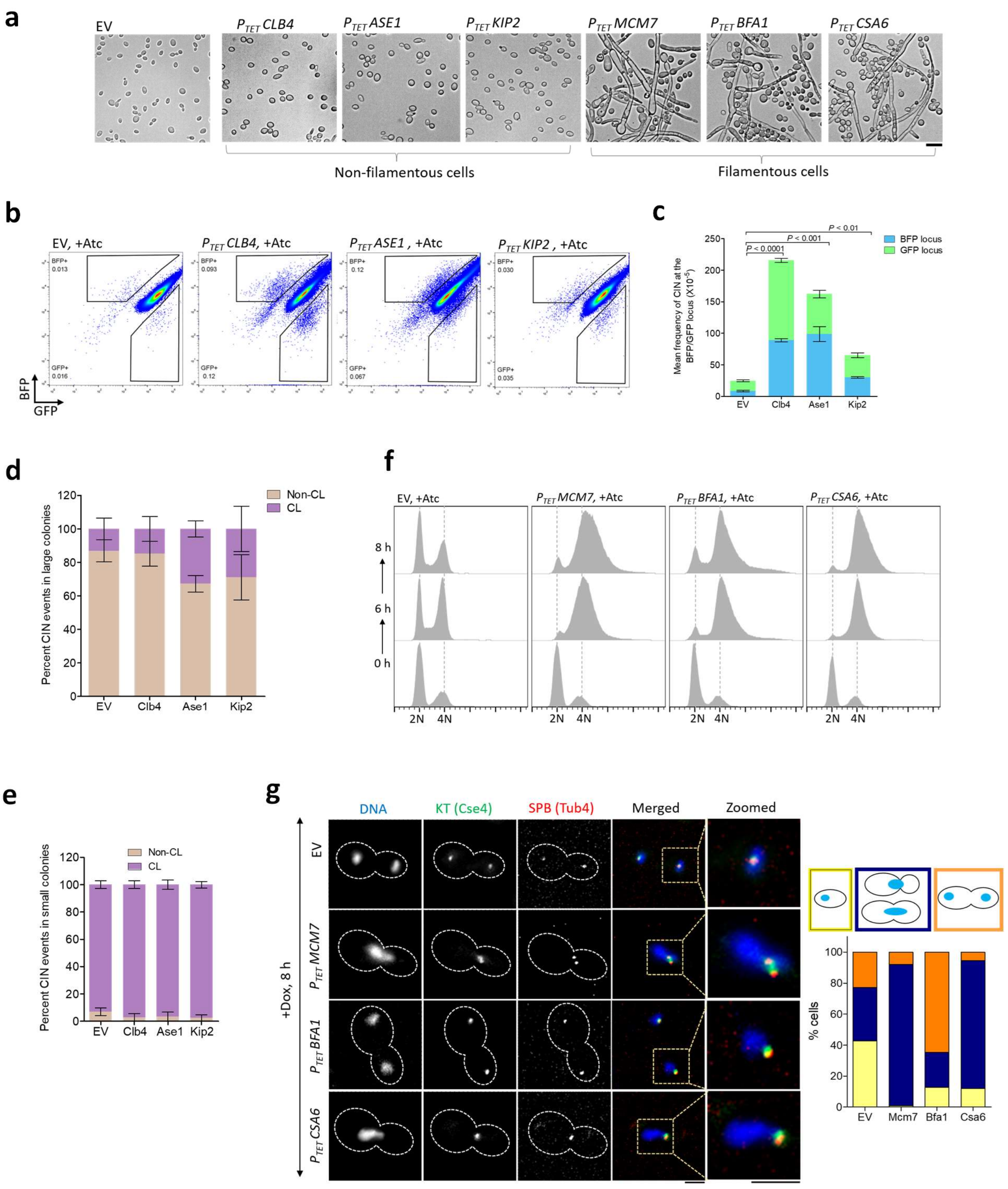

Supplementary Fig. 3. CIN associated with overexpression of *CSA* genes is regulated via distinct mechanisms. (a) Bright-field micrographs of the six overexpression strains, *CSA1*^{CLB4} (CaPJ152), *CSA2*^{ASE1} (CaPJ153), *CSA3*^{KIP2} (CaPJ154), *CSA4*^{MCM7} (CaPJ155), *CSA5*^{BFA1} (CaPJ156) and *CSA6* (CaPJ157), after 8 h of induction with Atc (3 µg/ml) and overnight

recovery in a rich medium without Atc. A representative image of EV (CaPJ160) is shown as a reference. Scale bar, 10 µm. **(b)** Representative BFP/GFP density plots of *CSA1*^{CLB4} (CaPJ152), *CSA2*^{ASE1} (CaPJ153) and *CSA3*^{KIP2} (CaPJ154), along with EV (CaPJ150), in presence of Atc (3 µg/ml). **(c)** Mean frequency of CIN (x10^{-5}) at the BFP/GFP locus in EV (CaPJ150) versus *CSA1*^{CLB4} (CaPJ152), *CSA2*^{ASE1} (CaPJ153) and *CSA3*^{KIP2} (CaPJ154) overexpression strains; N=3; mean ± SEM of three independent experiments is shown. Unpaired *t*-test, one-tailed, *P*-values show a significant difference; EV and Clb2, $P<0.0001$ for both BFP and GFP locus; EV and Ase1, $P=0.0008$ for both BFP and GFP locus; EV and Kip2, $P=0.0001$ and $P=0.0044$ for BFP and GFP locus, respectively. **(d)** Analysis of the marker genes, *ARG4*, *HIS1* and *HYG B* by replica plating large BFP$^-$GFP$^+$ colonies of EV (CaPJ150) and *CSA1*^{CLB4} (CaPJ152), *CSA2*^{ASE1} (CaPJ153) and *CSA3*^{KIP2} (CaPJ154) overexpression strains; n >300 colonies. The data represent the mean ± SEM of indicated number of colonies analyzed for each genotype from at least three independent experiments. **(e)** Marker analysis, as described previously, in small colonies of EV (n =102), *CSA1*^{CLB4} (n=40), *CSA2*^{ASE1} (n=30) and *CSA3*^{KIP2} (n=32) overexpression strains. The data represent the mean ± SEM of the indicated number of colonies analyzed for each genotype from three independent experiments. **(f)** Cell cycle analysis of EV (CaPJ160) and *CSA4*^{MCM7} (CaPJ165), *CSA5*^{BFA1} (CaPJ166) and *CSA6* (CaPJ167) overexpression strains; N=2. Briefly, overnight grown cells were induced for 8 h in presence of Atc (3 µg/ml). Cells were harvested and ethanol-fixed, treated with RNase, stained with propidium iodide and analyzed by flow cytometry for DNA content at specific time intervals. **(g)** *Left*, representative micrographs showing nuclear segregation and mitotic spindle in EV (CaPJ160) and *CSA4*^{MCM7} (CaPJ165), *CSA5*^{BFA1} (CaPJ166) and *CSA6* (CaPJ167) overexpression strains, after 8 h of growth in presence of Dox (50 µg/ml). The nuclear division was analyzed by both Hoechst staining as well as by localization of a KT protein, Cse4-GFP. The spindle integrity was analyzed using Tub4-mCherry, an SPB protein, as a marker. Scale bar, 3 µm. *Right*, quantitation of the cells with indicated phenotypes. n ≥100 cells. Source data are provided as a Source Data file.

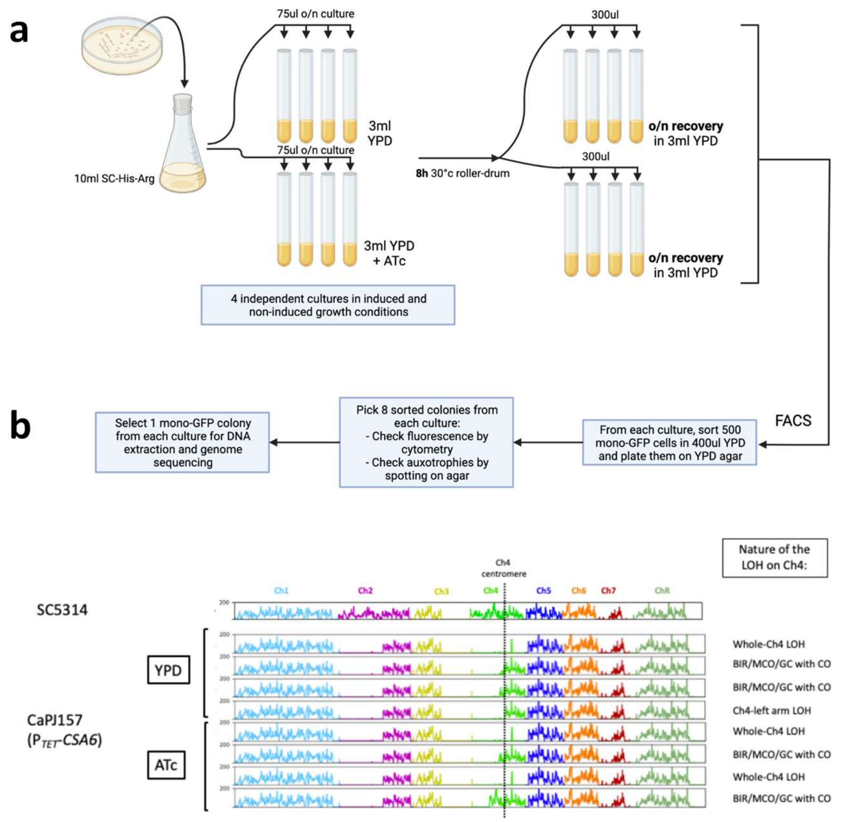

Supplementary Fig. 4. Analysis of extent of genome instability upon *CSA6* overexpression. **(a)** Experimental design to select cells having undergone LOH on the left arm of Ch4, and that were further processed for DNA extraction and genome sequencing. **(b)** Plots showing the density in heterozygous SNPs across 10000kb windows along the eight chromosomes in $CSA6^{OE}$ strain (treated with or without Atc-3µg/ml), along with the reference strain SC5314. Sequencing confirms the presence of homozygosis on the left arm of Ch4 in $BFP^{-}GFP^{+}$ sorted cells. Additional LOH events, such as LOH on Ch2, have previously been described in the parental strain [19]. The black vertical dotted line shows the position of the Ch4 centromere. The extent of LOH provides information on the molecular mechanism at the origin of the LOH. BIR, break-induced replication; MCO, mitotic crossing over; GC, gene conversion.

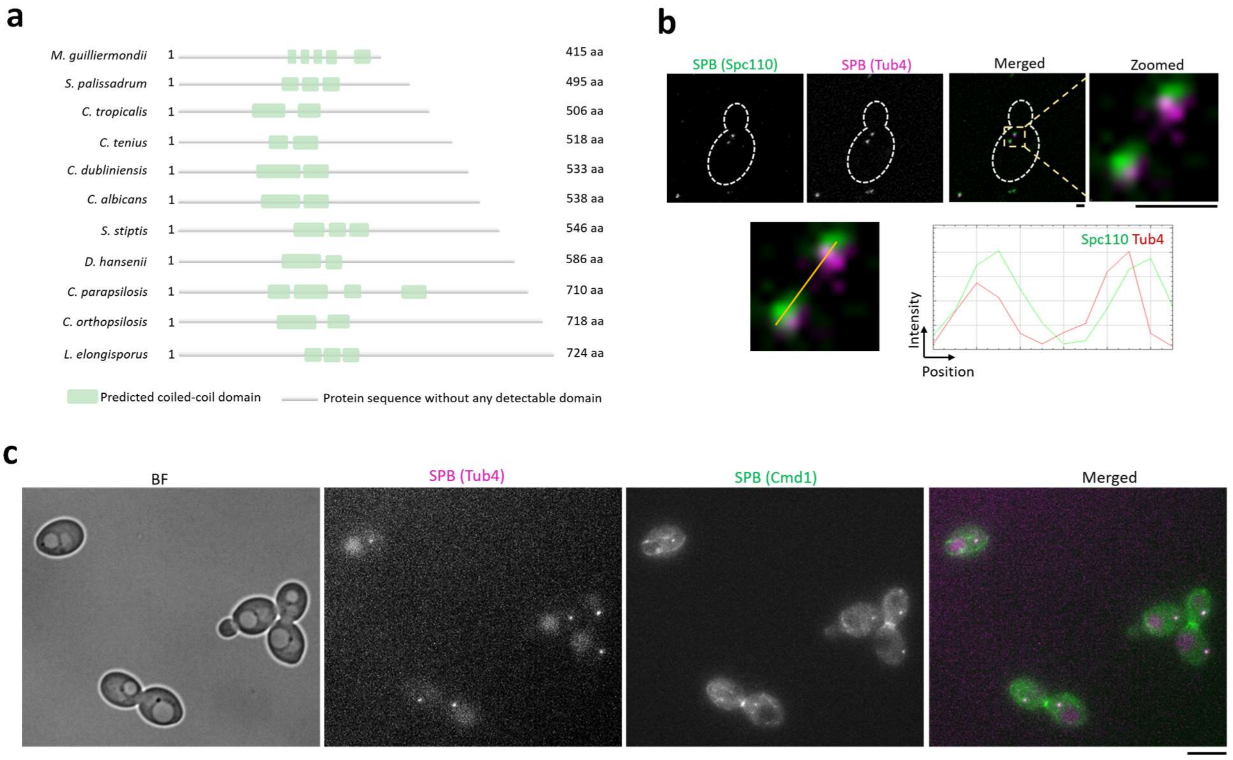

Supplementary Fig. 5. Analysis of the Csa6 domain architecture and sub-cellular localization of Spc110 and Cmd1 in *C. albicans*. (a) Schematic illustrating the protein domain architecture alignment of Csa6 in the indicated fungal species. Length of the protein is mentioned as amino acids (aa). Approximate positions of the predicted coiled-coil domain, identified using HMMER [20] phmmer searches, is shown. **(b)** *Top*, micrograph showing SPB localization of Spc110 in CaPJ123. Tub4mCherry is used as a SPB marker. Scale bar, 1 µm. *Bottom*, histogram plot showing the fluorescence intensity profile of Tub4-mCherry with Spc110-GFP across the indicated line. **(c)** Sub-cellular localization of Cmd1 in CaPJ124 at various cell cycle stages. SPBs are marked with Tub4mCherry for reference. BF, brightfield. Scale bar, 5 µm.

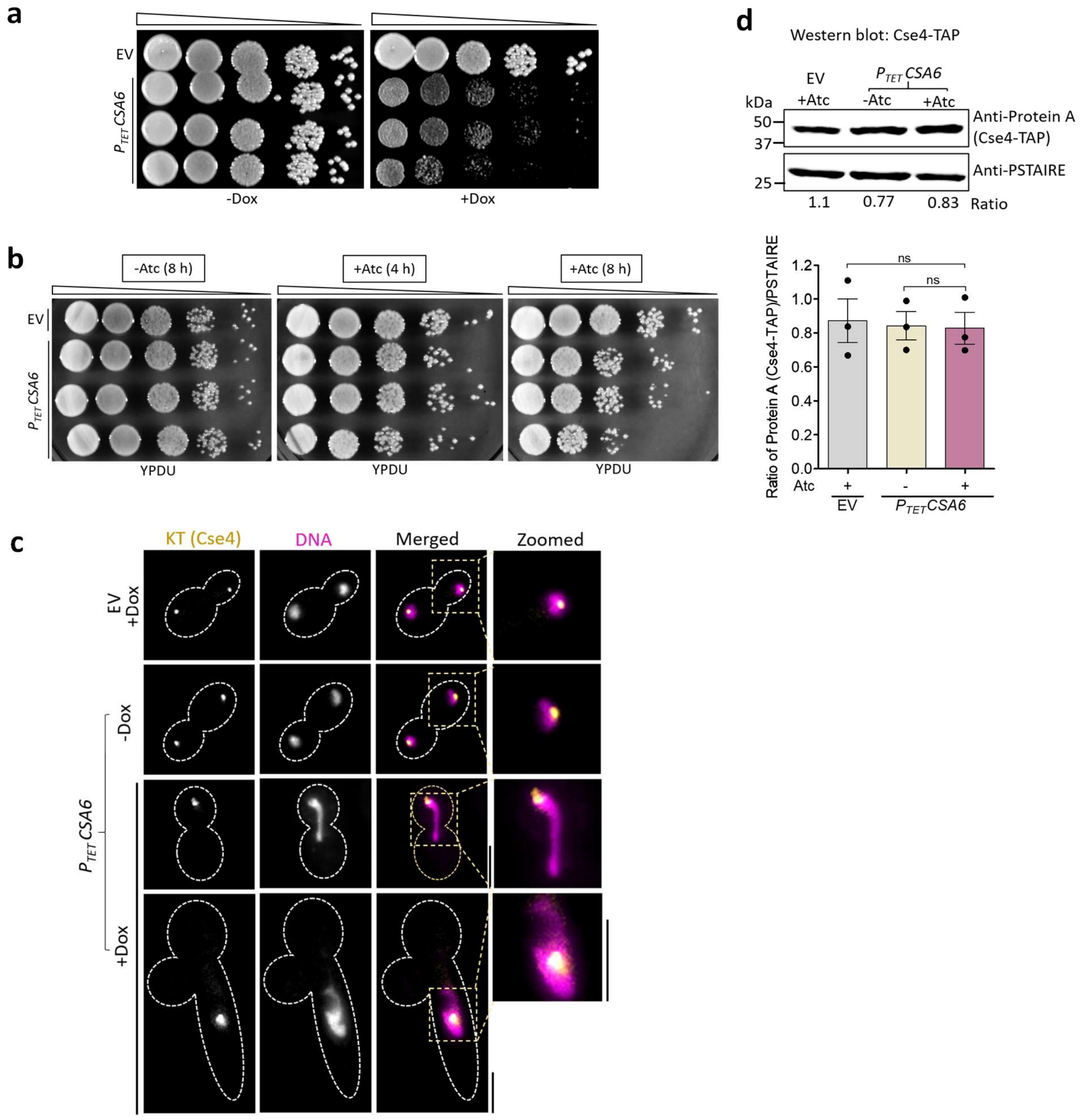

Supplementary Fig. 6. Overexpression of *CSA6* affects cell growth and cell viability but does not perturb kinetochore integrity in *C. albicans*. (a) Ten-fold serial dilutions, starting from 10^5 cells, each of CaPJ170 (EV) or CaPJ176 (*CSA6* ^{OE}) were spotted on YPDU agar plates with or without Dox (50 µg/ml) and incubated at 30°C for two days. **(b)** Analysis of cell viability in the *CSA6* ^{OE} strain. Ten-fold serial dilutions, starting from 10^5 cells, each of CaPJ170 (EV) and CaPJ176 (*CSA6* ^{OE}) were spotted on YPDU agar plates after growing the strains in presence or

absence of Atc (3 µg/ml) for indicated time period. **(c)** Localization of Cse4-GFP in CaPJ183 (*CSA6* ^{OE} mutant) and CaPJ182 (EV), after 8 h of growth under indicated conditions of Dox (50 µg/ml). The nucleus was stained with Hoechst dye for reference. Scale bar, 5 µm. **(d)** *Top*, immunoblot analysis of Cse4-TAP levels in CaPJ173 (EV) and CaPJ179 (*CSA6*^{OE} mutant) in presence or absence of Atc (3 µg/ml) using anti-Protein A antibodies; *N*=3. Mean ± SEM of three independent experiments is indicated. PSTAIRE was used as a loading control. Cse4-TAP levels were normalized by calculating the ratio of Protein A/PSTAIRE. *Bottom*, Quantitation of the normalized Cse4-TAP levels; *N*=3. One-way ANOVA and Bonferroni posttest, *P*-values were non-significant (ns) (>0.05). Source data are provided as a Source Data file.

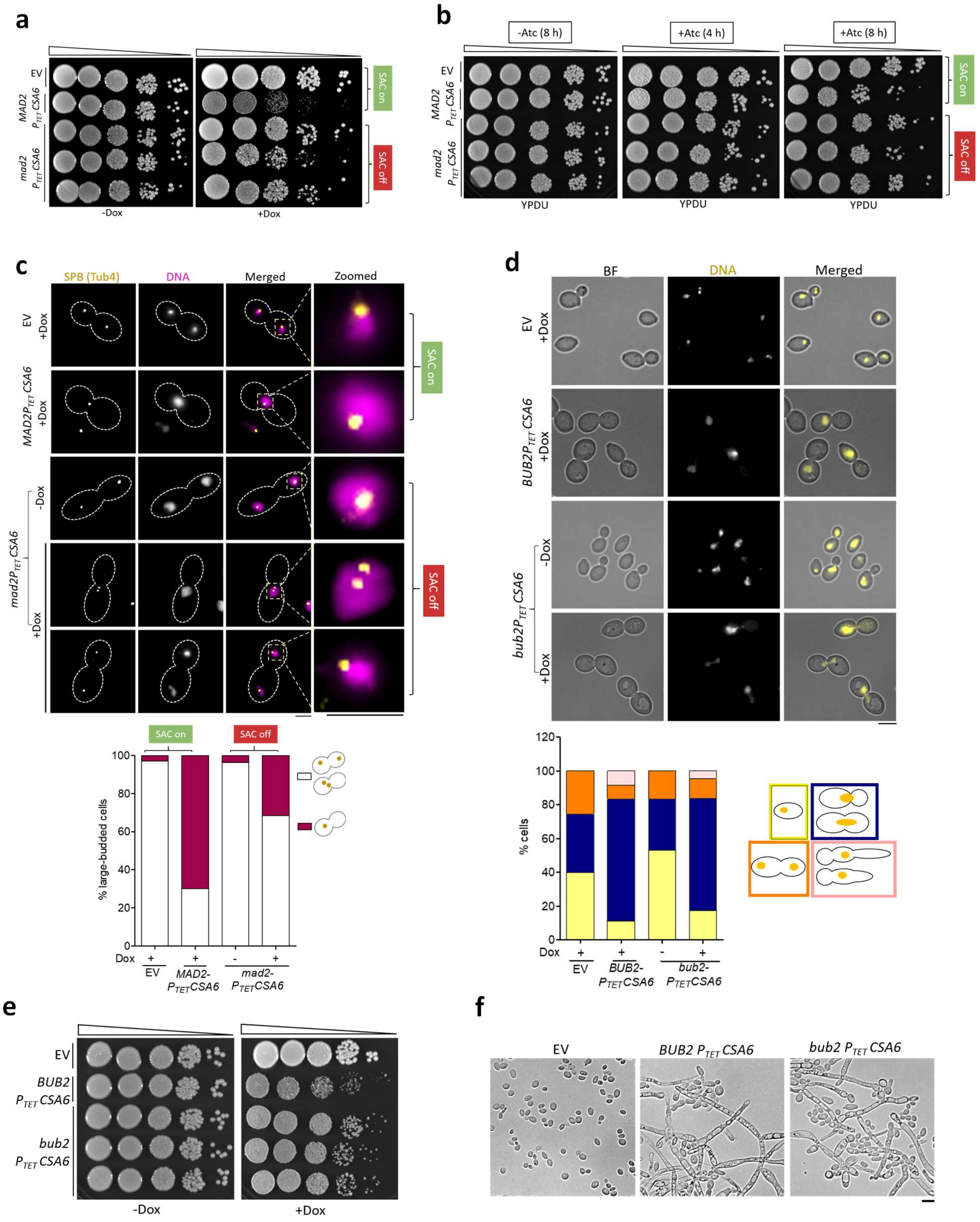

Supplementary Fig. 7. ***CSA6*** **over-expression associated G2/M arrest is relieved upon** ***mad2*** **but not** ***bub2*** **deletion. (a)** Spot dilution analysis of CaPJ170 (EV), CaPJ176 (*MAD2CSA6* ^{OE})

and CaPJ197 (*mad2CSA6*^{OE}). Ten-fold serial dilutions, starting from 10^5 cells, were spotted on YPDU agar plates with or without Dox (50 µg/ml) and incubated at 30°C for two days. **(b)** Analysis of cell viability in the *CSA6* ^{OE} strain when the SAC is inactivated. Ten-fold serial dilutions, starting from 10^5 cells, each of CaPJ170 (EV), CaPJ176 (*MAD2CSA6* ^{OE}) and CaPJ197 (*mad2CSA6*^{OE}) were spotted on YPDU agar plates after growing the strains in presence or absence of Atc (3 µg/ml) for the indicated time. **(c)** *Top,* localization patterns of Tub4-GFP in large-budded cells of CaPJ171 (EV), CaPJ177 (*MAD2CSA6*^{OE}) and CaPJ198 (*mad2CSA6* ^{OE}) after 8 h of growth under indicated conditions of Dox (50 µg/ml). Hoechst staining was done to mark the nuclei. Scale bar, 3 µm. *Bottom*, quantitation of the large-budded cells with the indicated Tub4 phenotypes; $n \geq 100$ cells. **(d)** *Top*, representative images of Hoechst-stained CaPJ170 (EV), CaPJ176 (*BUB2CSA6*^{OE}) and CaPJ200 (*bub2CSA6*^{OE}) after 8 h of growth under indicated conditions of Dox (50 µg/ml). Scale bar, 5 µm. *Bottom*, percent cells with indicated cell types; $n \geq 100$ cells. **(e)** Ten-fold serial dilutions, starting from 10^5 cells, each of CaPJ170 (EV), CaPJ176 (*BUB2CSA6*^{OE}) and CaPJ200 (*bub2CSA6*^{OE}) were spotted on YPDU agar plates with or without Dox (50 µg/ml) and incubated at 30°C for two days. **(f)** Brightfield micrographs of CaPJ170 (EV), CaPJ176 (*BUB2CSA6*^{OE}) and CaPJ200 (*bub2CSA6*^{OE}) from the agar plates shown in *E*. Scale bar, 10 µm.

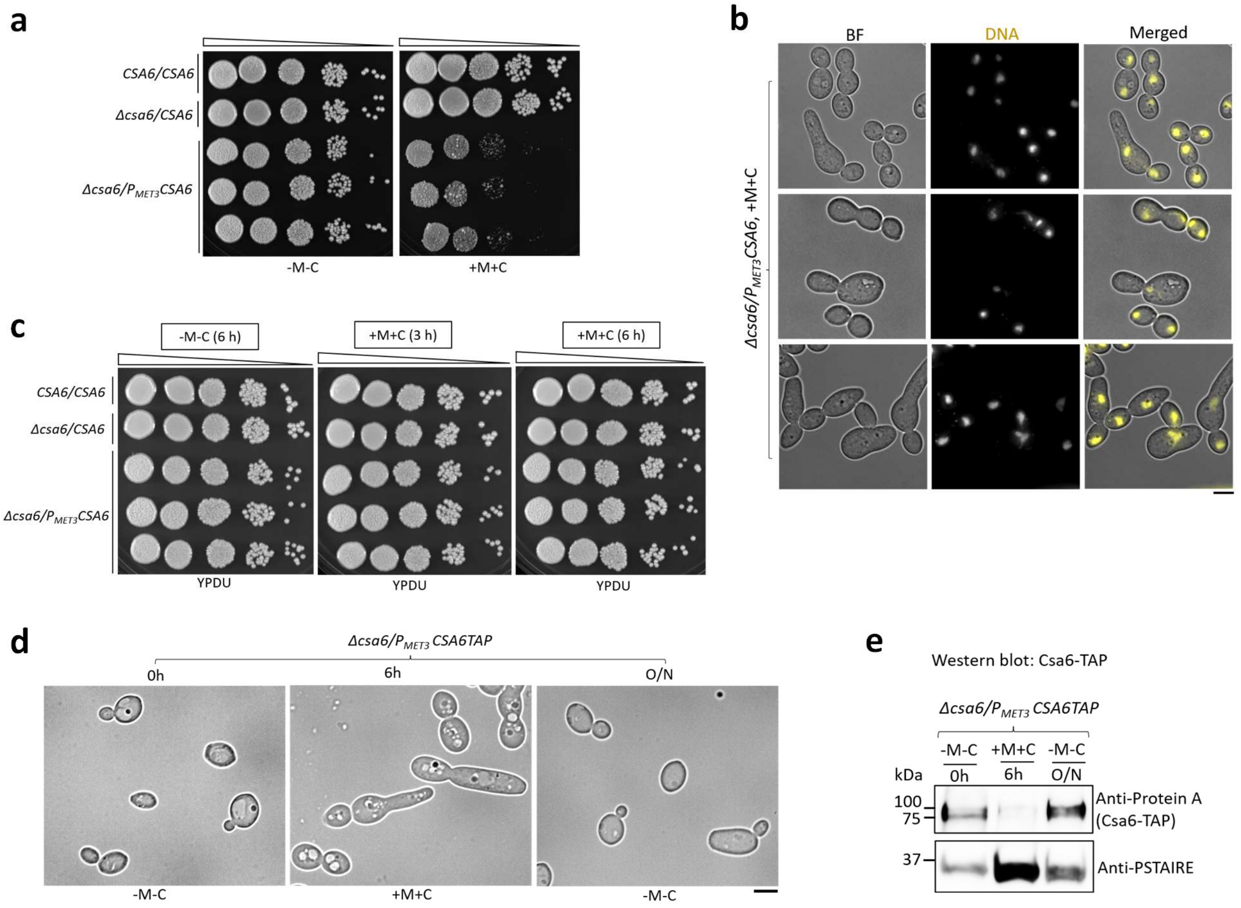

Supplementary Fig. 8. Csa6 is essential for viability and its depletion causes a reversible late anaphase/telophase arrest. (a) Spot dilution analysis of SN148 (*CSA6/CSA6*), CaPJ209 (*csa6/CSA6*) and CaPJ210 (*CSA6^{PSD}*). Ten-fold serial dilutions, starting from 10^5 cells, were spotted on agar plates with permissive (YPDU-Met-Cys) or repressive (YPDU + 5 mM Met and 5 mM Cys) media and incubated at 30°C for two days. **(b)** Representative micrograph showing aberrant cell morphology of Hoechst stained CaPJ210 (*CSA6^{PSD}*) after 6 h of growth in repressive media. Scale bar, 5 µm. **(c)** Analysis of cell viability in *CSA6 ^{PSD}* strain. Ten-fold serial dilutions, starting from 10^5 cells, each of SN148 (*CSA6/CSA6*), CaPJ209 (*csa6/CSA6*) and CaPJ210 (*CSA6^{PSD}*) were spotted on YPDU agar plates after growing the strains in permissive or repressive media for indicated time period. **(d)** Representative brightfield micrographs showing reversal of cell cycle arrest phenotype in the *CSA6^{PSD}* mutant. Briefly, overnight grown culture of *CSA6^{PSD}* strain (CaPJ210) was reinoculated in repressive media for 6 h after which it was shifted to permissive media and allowed to grow overnight (O/N). Scale bar, 5 µm. **(e)** Western blot

analysis using anti-Protein A antibodies to compare Csa6 levels in *CSA6*^{PSD} strain (CaPJ212) when shifted from repressive media (for 6 h) to permissive media (O/N); *N*=3. PSTAIRE was used as a loading control. Source data are provided as a Source Data file.

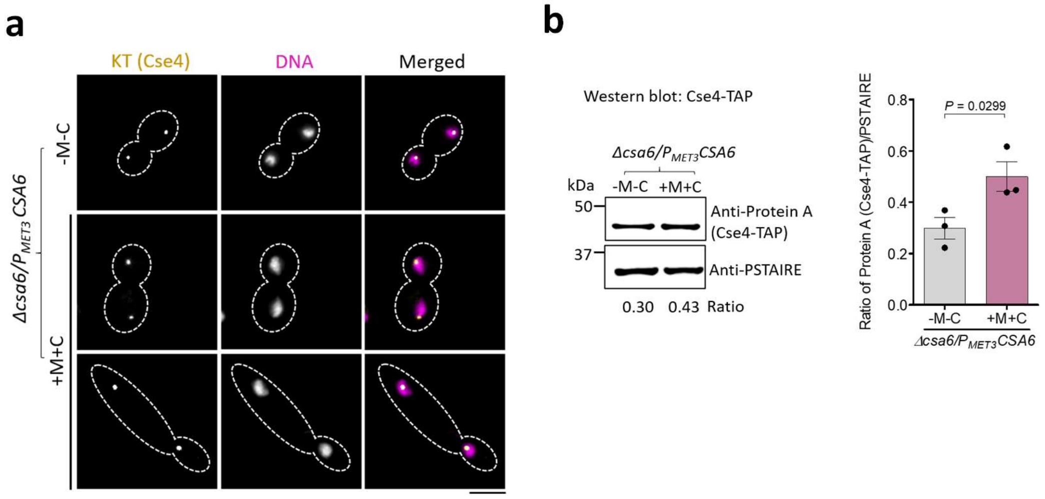

Supplementary Fig. 9. Csa6 depleted cells duplicate and segregate their nuclei. (a) Localization of Cse4-GFP in *CSA6*^{PSD} strain CaPJ213, after 6 h of growth in permissive (YPDU-M-C) or repressive (YPDU + 5 mM M and 5 mM C) conditions. Cse4-GFP colocalized with the nucleus, stained with Hoechst dye. Scale bar, 5 µm. **(b)** *Left*, western blot analysis using anti-Protein A antibodies to compare Cse4-TAP levels in *CSA6*^{PSD} strain CaPJ214 when grown under permissive (YPDU-M-C) or repressive (YPDU + 5 mM M and 5 mM C) conditions for 6 h; *N*=3. PSTAIRE was used as a loading control. Cse4-TAP levels were normalized by calculating the ratio of Protein A/PSTAIRE. *Right*, quantitation of the normalized Cse4 levels; *N*=3; mean ± SEM of three independent experiments is indicated. Paired *t*-test, two-tailed, *P*-value shows a significant difference (*P*=0.0299). Source data are provided as a Source Data file.

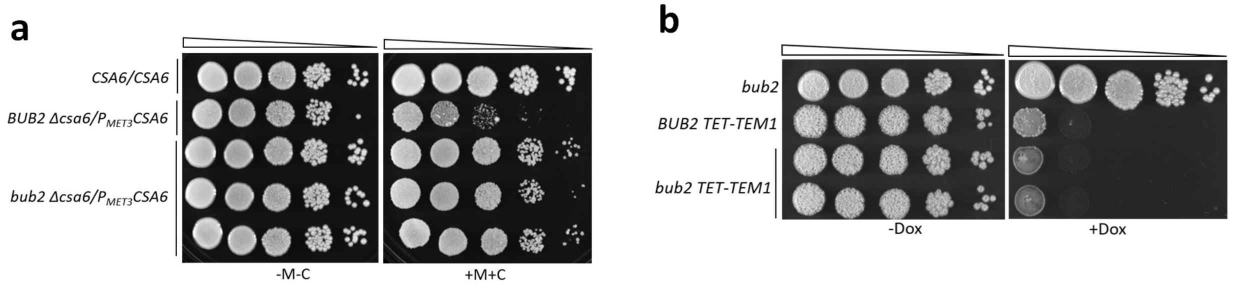

Supplementary Fig. 10. Deletion of Bub2 rescues the growth defect of the *CSA6^PSD* mutant. **(a-b)** Ten-fold serial dilutions, starting from 10^5 cells, of indicated genotypes were spotted on either YPDU or YPDU containing 5 mM methionine (M) and cysteine (C) or doxycycline (20µg/ml) followed by incubation at 30°C for two days.

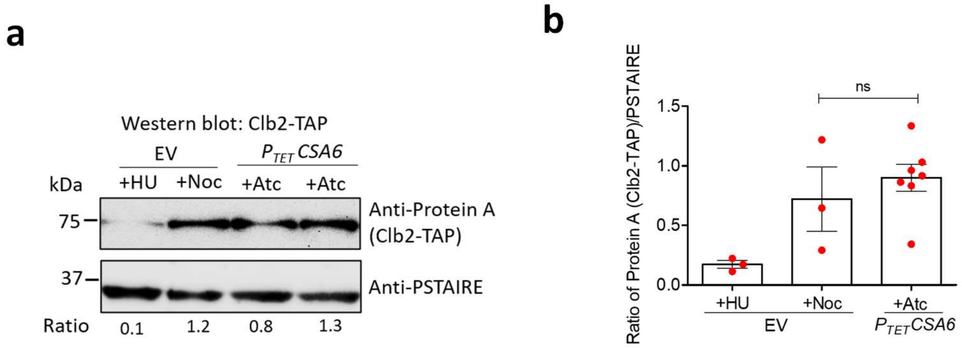

Supplementary Fig. 11. Csa6 overexpression does not affect Clb2 levels. (a) Western blot analysis using anti-Protein A antibodies to compare Clb2-TAP levels in EV (CaPJ221) treated with hydroxyurea (HU) or nocodazole (Noc) and *CSA6*^{OE} strain (CaPJ222) grown in presence of Atc (3 µg/ml) for 8 h; *N*=3. For HU/Noc treatment, cells were grown in YPDU containing either HU (200mM) or Noc (20 µg/ml) for 4 h. PSTAIRE was used as a loading control. Clb2-TAP levels were normalized by calculating the ratio of Protein A/PSTAIRE. **(b)** Quantitation of the normalized Clb2 levels; *N* ≥3. The data represent the mean ± SEM of at least three independent experiments. Unpaired *t*-test, two-tailed, *P*-value was non-significant (ns) (>0.05). Source data are provided as a Source Data file.

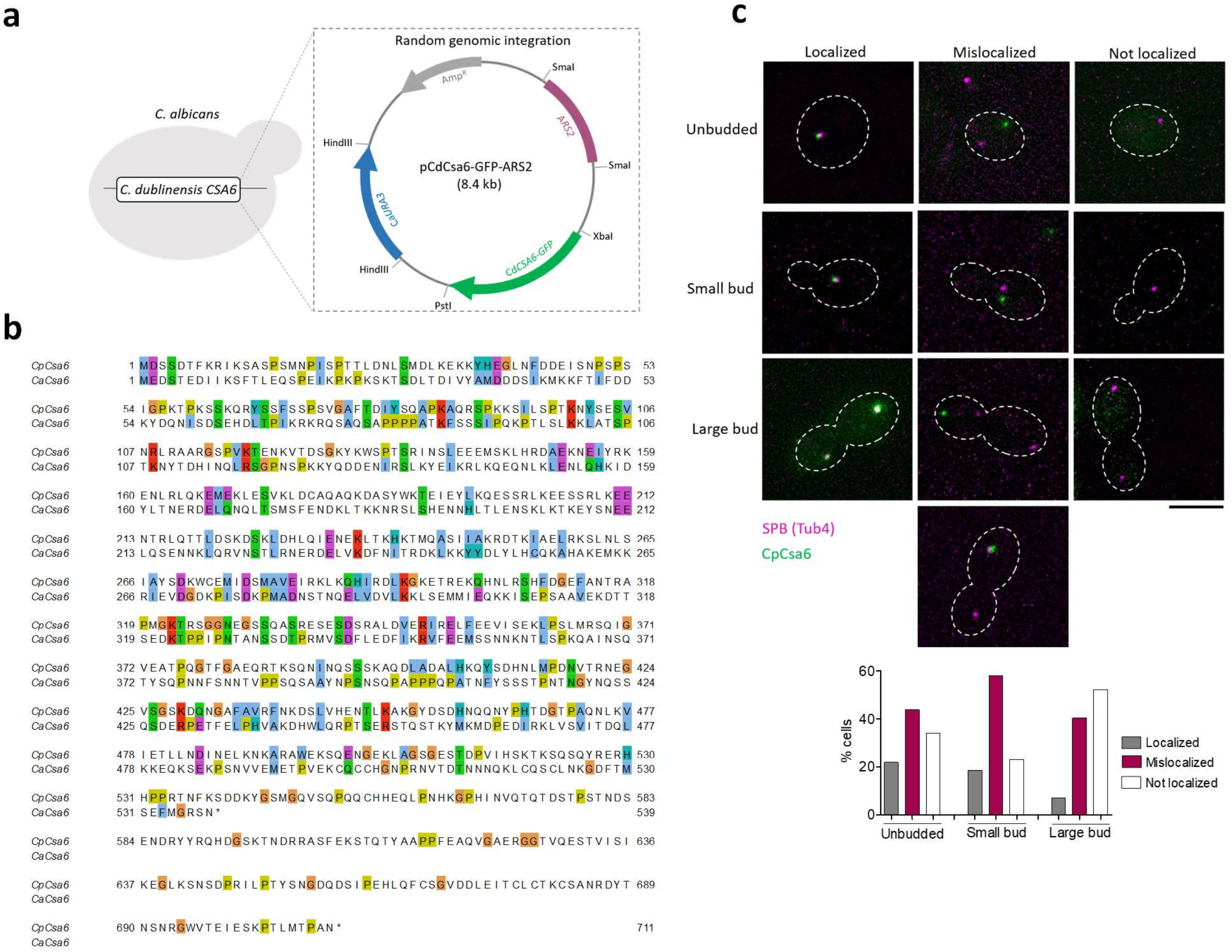

Supplementary Fig. 12. Ectopic expression of CdCsa6 and CpCsa6 in *C. albicans.* (a) Ectopic expression of CdCsa6 in *C. albicans* by random genomic integration of the ARS-containing plasmid. Vector map of pCdCsa6-GFP-ARS2 depicts the cloned sites of Ca*URA3*, Ca*ARS2* and Cd*CSA6-GFP*. The Cd*CSA6-GFP* fragment contains the GFP tag, Cd*CSA6* (*ORF Cd36_16290*) without the stop codon and the promoter region of Cd*CSA6*. **(b)** Pair-wise alignment of amino acid sequences of CpCsa6 and CaCSa6 using Clustal Omega and Jalview. **(c)** *Top*, micrographs showing sub-cellular localization of CpCsa6 in CaPJ304 (*CSA6*^{PSD} expressing *CpCSA6*) under permissive conditions (YPDU-Met-Cys). Scale bar, 5 µm. CpCsa6 either localizes to the SPB (localized), shows localization elsewhere in the cell (mislocalized) or does not show any detectable fluorescence (not localized). *Bottom*, quantitation of various types of CpCsa6 localization in unbudded, small budded and large budded cells; *n* ≥40 cells for each cell type.

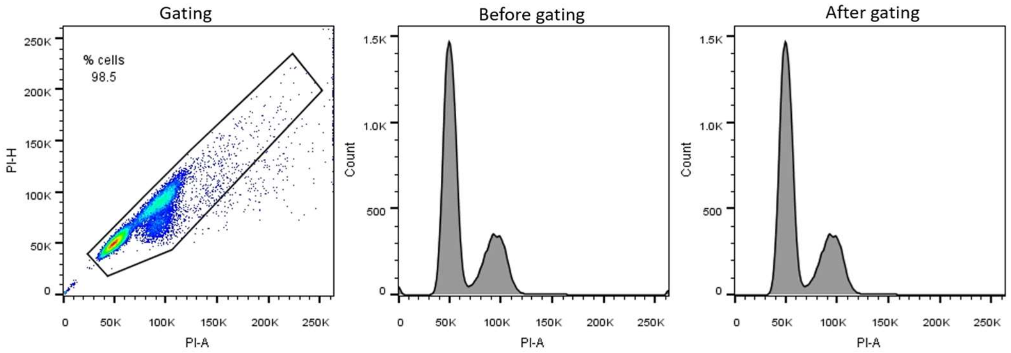

Supplementary Fig. 13. Gating strategy applied to all flow cytometric cell cycle data of this paper. Distribution of DNA content in EV after 8 h of growth in presence of Atc (3 µg/ml) before gating and after applying the indicated gate.

Supplementary Table 1. Quantification of BFP/GFP loss frequency in EV

Sample	Frequency of BFP^+GFP^- cells ($x10^{-5}$)	Frequency of BFP^- GFP^+ cells ($x10^{-5}$)
1	7.14	19
2	7.52	24
3	13	16
4	5.7	24
5	6.69	20
6	7.75	15
7	24	16
8	18	15
9	15	22
10	13	20
11	15	21
12	24	19
13	7.8	21
14	15	22
15	14	15
16	11	14
17	15	13
18	20	16
19	14	15
20	22	20
21	18	25
22	15	21
Mean	14.02	18.77

Supplementary Table 2. BFP/GFP loss frequency in the primary hits

ORF no.	Orthologs in *S. cerevisiae*	Frequency of $BFP^{+}GFP^{-}$ cells ($x10^{-5}$)	Fold change	Frequency of $BFP^{-}GFP^{+}$ cells ($x10^{-5}$)	Fold change
19.1447	-	180	13.0	210	11.2
19.7186	*CLB4*	180	13.0	180	9.6
19.608	*BFA1*	140	10.0	190	10.2
19.3135	*UBX2*	120	8.6	45	2.4
19.202	*MCM7*	82	5.9	120	6.4
19.1048	*IFD6*	63	4.5	83	4.4
19.6588	*NBP2*	83	5.9	40	2.1
19.1601	*RPL3*	70	5.0	43	2.3
19.3437	-	72	5.1	37	2.0
19.1934	*HST3*	66	4.7	39	2.0
19.1542	*HEX3*	50	3.6	54	2.9
19.6778	*DRS2*	43	3.0	55	2.9
19.4153	*ULA1*	52	3.7	42	2.2
19.1396	*AGE2*	53	3.8	39	2.0
19.3349	*RPB2*	38	2.7	44	2.3
19.1747	*KIP2*	41	2.9	50	2.7
19.4979	*KNS1*	40	2.8	55	2.9
19.7377	*ASE1*	40	2.9	48	2.6
19.1999	-	33	2.3	44	2.3
19.3421.1	*ROX3*	35	2.5	47	2.5
19.6118	*DSS4*	38	2.7	39	2.1
19.4340.1	*SMX3*	33	2.4	39	2.1
19.5212	*CST9*	32	2.3	36	1.9

Supplementary Table 3. Strains used in this study

Name (Description)	Genotype	Reference
SN148	*Δura3::imm434/Δura3::imm434, Δhis1::hisG/Δhis1::hisG, Δarg4::hisG/Δarg4::hisG, Δleu2::hisG/Δleu2::hisG*	[21]
YJB8675	*Δura3::imm434/Δura3::imm434, Δhis1::hisG/Δhis1::hisG, Δarg4::hisG/Δarg4::hisG, CSE4-GFPCSE4/CSE4*	[22]
J110	SN148 *mad2::ARG4/mad2::LEU2*	[13]
CEC3867	SN148 *Ca21ch4_C_albicans_SC5314:473390 to 476401Δ::PTDH3-GFP-ARG4/Ca21ch4_C_albicans_SC5314:473390 to 476401Δ::PTDH3-BFP-HIS1, ADH1/adh1::PTDH3-cartTA-SAT1*	[5]
CAKS102	SN148 *CSE4/CSE4-TAP::URA3*	[12]
Cd36 (*C. dubliniensis* prototroph)	*URA3/URA3* (clinical isolate)	[11]
MYA-3404 (*C. tropicalis* prototroph)	Clinical isolate	[8]
SBC169	As THE1 but *URA3-TETp-TEM1/tem1*Δ*::dpl200*	[23]
C. parapsilosis	Accession number NRRL Y-8312	USDA
CEC5201 (CSA reporter)	CEC3867 *Ca22ch4_C_albicans_SC5314:1452840 to 1453029*Δ*::*P_{TDH3}*-RFP-Hyg*^{R}*/Ca22ch4_C_albicans_SC5314:1452840 to 1453029*	This study
CaPJ148 (Mono-BFP)	*SN148 Ca21ch4_C_albicans_SC5314:473390 to 476401Δ::PTDH3-BFP-HIS1/Ca21ch4_C_albicans_SC5314:473390 to 476401*	This study
CaPJ149 (Mono-GFP)	*SN148 Ca21ch4_C_albicans_SC5314:473390 to 476401*Δ*::PTDH3-GFP-ARG4/Ca21ch4_C_albicans_SC5314:473390 to 476401*	This study
CaPJ150 (EV in CSA reporter)	CEC5201 *RPS1/RPS1::PTET-GtwB-URA3*	This study
CaPJ151 (*CDC20*^{OE} in CSA reporter)	CEC5201 *RPS1/RPS1::PTET-CDC20-URA3*	This study
CaPJ152 (*CSA1*^{CLB4} overexpression in CSA reporter)	CEC5201 *RPS1/RPS1::PTET-CLB4-URA3*	This study

CaPJ153 (*CSA2*^{ASE1} overexpression in CSA reporter)	CEC5201 *RPS1/RPS1::PTET-ASE1-URA3*	This study
CaPJ154 (*CSA3*^{KIP2} overexpression in CSA reporter)	CEC5201 *RPS1/RPS1::PTET-KIP2-URA3*	This study
CaPJ155 (*CSA4*^{MCM7} overexpression in CSA reporter)	CEC5201 *RPS1/RPS1::PTET-MCM7-URA3*	This study
CaPJ156 (*CSA5*^{BFA1} overexpression in CSA reporter)	CEC5201 *RPS1/RPS1::PTET-BFA1-URA3*	This study
CaPJ157 (*CSA6*^{OE} in CSA reporter)	CEC5201 *RPS1/RPS1::PTET-CSA6-URA3*	This study
CaPJ158	YJB8675 *ADH1/adh1::PTDH3-cartTA-SAT1*	This study
CaPJ159	CaPJ158 *TUB4/TUB4-mCherry::ARG4*	This study
CaPJ160 (EV in *CSE4-GFP, TUB4-mCherry*)	CaPJ159 *RPS1/RPS1::PTET-GtwB-URA3*	This study
CaPJ165 (*CSA4*^{MCM7} overexpression in *CSE4-GFP, TUB4-mCherry*)	CaPJ159 *RPS1/RPS1::PTET-MCM7-URA3*	This study
CaPJ166 (*CSA5*^{BFA1} overexpression in *CSE4-GFP, TUB-4mCherry*)	CaPJ159 *RPS1/RPS1::PTET-BFA1-URA3*	This study
CaPJ167 (*CSA6*^{OE} in *CSE4-GFP, TUB4-mCherry*)	CaPJ159 *RPS1/RPS1::PTET-CSA6 -URA3*	This study
CaPJ169	SN148 *ADH1/adh1::PTDH3-cartTA-SAT1*	This study
CaPJ180 (*CSA6-TAP*)	SN148 *CSA6/CSA6-TAP::ARG4*	This study
CaPJ181 (*PTETCSA6-TAP*)	CaPJ169 *RPS1/RPS1::PTET-CSA6-TAP-URA3*	This study

CaPJ170 (EV in SN148)	CaPJ169 *RPS1/RPS1::PTET-GtwB-URA3*	This study
CaPJ176 (*CSA6*^{OE} in SN148)	CaPJ169 *RPS1/RPS1::PTET-CSA6-URA3*	This study
CaPJ162	YJB8675 *TUB1/TUB1-mCherry::HIS1*	This study
CaPJ163	CaPJ162 *ADH1/adh1::PTDH3-cartTA-SAT1*	This study
CaPJ182 (EV in *CSE4-GFP*)	CaPJ163 *RPS1/RPS1::PTET-GtwB-URA3*	This study
CaPJ183 (*CSA6*^{OE} in *CSE4-GFP*)	CaPJ163 *RPS1/RPS1::PTET-CSA6-URA3*	This study
CaPJ173 (*CSE4-TAP* in EV)	CaPJ170 *CSE4/CSE4-TAP::LEU2*	This study
CaPJ179 (*CSE4-TAP* in *CSA6*^{OE})	CaPJ176 *CSE4/CSE4-TAP::LEU2*	This study
CaPJ171 (EV in *TUB4-GFP*)	CaPJ170 *TUB4/TUB4-GFP:: HIS1*	This study
CaPJ172 (EV in *TUB4-GFP, TUB1-mCherry*)	CaPJ170 *TUB4/TUB4-GFP:: HIS1, TUB1/TUB1-mCherry::ARG4*	This study
CaPJ177 (*CSA6*^{OE} in *TUB4-GFP*)	CaPJ176 *TUB4/TUB4-GFP:: HIS1*	This study
CaPJ178 (*CSA6*^{OE} in *TUB4-GFP, TUB1-mCherry*)	CaPJ177 *TUB1/TUB1-mCherry::ARG4*	This study
CaPJ196	CaJ110 *ADH1/adh1::PTDH3-cartTA-SAT1*	This study
CaPJ197 (*CSA6*^{OE} in *mad2*)	CaPJ196 *RPS1/RPS1::PTET-CSA6-URA3*	This study
CaPJ198 (*CSA6*^{OE} in *mad2, TUB4-GFP)*	CaPJ197 *TUB4/TUB4-GFP:: HIS1*	This study
CaPJ109	SN148 *bub2::FRT/BUB2*	This study
CaPJ110	SN148 *bub2::FRT/ bub2::FRT*	This study
CaPJ199	CaPJ110 *ADH1/adh1::PTDH3-cartTA-SAT1*	This study
CaPJ200 (*CSA6*^{OE} in *bub2*)	CaPJ199 *RPS1/RPS1::PTET-CSA6-URA3*	This study
CaPJ209 (*CSA6* heterozygous null in SN148)	SN148 *csa6::FRT/CSA6*	This study

CaPJ210 (*$CSA6^{PSD}$* in SN148)	SN148 *csa6::FRT/MET3prCSA6::URA3*	This study
CaPJ212 (*P_{MET3}CSA6-TAP*)	SN148 *csa6::FRT/MET3prCSA6-TAP-ARG4::URA3*	This study
CaPJ113	YJB8675 *csa6::FRT/CSA6*	This study
CaPJ213 (*$CSA6^{PSD}$* in *CSE4-GFP*)	YJB8675 *csa6::FRT/MET3prCSA6::URA3*	This study
CaPJ214 (*CSE4-TAP* in *$CSA6^{PSD}$*)	CaPJ210 *CSE4/CSE4-TAP::LEU2*	This study
CaPJ211 (*$CSA6^{PSD}$* in *TUB4-GFP, TUB1-mCherry*)	CaPJ210, *TUB4/TUB4-GFP:: HIS1, TUB1/TUB1-mCherry::ARG4*	This study
CaPJ216 (*SOL1-TAP*)	SN148 *SOL1/SOL1-TAP::HIS1*	This study
CaPJ217 (*P_{TET}SOL1-TAP*)	CaPJ209 *ADH1/adh1::PTDH3-cartTA-SAT1 RPS1/RPS1::PTET-SOL1-TAP-URA3*	This study
CaPJ215 (*$CSA6^{PSD}$* in *$SOL1^{OE}$*)	SN148 *csa6::FRT/MET3prCSA6::HIS1, ADH1/adh1::PTDH3-cartTA-SAT1, RPS1/RPS1::PTET-SOL1-URA3*	This study
CaPJ218 (*TEM1-GFP* in *$CSA6^{PSD}$*)	SN148 *csa6::FRT/MET3prCSA6::URA3, TUB4/TUB4-mCherry::NAT, TEM1/TEM1-GFP::HIS1*	This study
CaPJ219	CaPJ210 *bub2::FRT/ bub2::FRT*	This study
CaPJ220	SBC169 *bub2::FRT/ bub2::FRT-NAT-FRT*	This study
CaPJ221	CaPJ170 *CLB2/CLB2-TAP::HIS1*	This study
CaPJ222	CaPJ176 *CLB2/CLB2-TAP::HIS1*	This study
CaPJ119 (*CSA6-mCherry* in *CSE4-GFP*)	YJB8675 *CSA6/CSA6-mCherry::ARG4*	This study
CaPJ117	SN148 *csa6::FRT/ CSA6-mCherry::ARG4*	This study
CaPJ118	SN148 *CSA6/CSA6-mCherry::ARG4*	This study
CaPJ120 (*CSA6-mCherry* in *TUB4-GFP*)	CaPJ118 *TUB4/TUB4-GFP::HIS1*	This study
CaPJ121 (*CSA6-mCherry* in *SPC110-GFP*)	CaPJ118 *SPC110/SPC110-GFP::HIS1*	This study
CaPJ122 (*CSA6-mCherry* in *CMD1-GFP*)	CaPJ118 *CMD1/CMD1 -GFP::HIS1*	This study

CaPJ123	SN148 *TUB4/TUB4-mCherry::NAT, SPC110/SPC110-GFP::HIS1*	This study
CaPJ124	SN148 *TUB4/TUB4-mCherry::NAT, CMD1/CMD1 -GFP::HIS1*	
CaPJ300	CaPJ209 *TUB4/TUB4-mCherry::NAT + pCdCSA6-GFP-ARS2::URA3*	This study
CaPJ301	SN148 *csa6::FRT/MET3prCSA6::HIS1, TUB4/TUB4-mCherry::NAT*	This study
CaPJ302	SN148 *csa6::FRT/MET3prCSA6::HIS1, TUB4/TUB4-mCherry::NAT + pCdCSA6-GFP-ARS2::URA3*	This study
CaPJ303	SN148 *csa6::FRT/MET3prCSA6::HIS1, TUB4/TUB4-mCherry::NAT RPS1/RPS1:: CtCSA6-GFP::URA3*	This study
CaPJ304	SN148 *csa6::FRT/MET3prCSA6::HIS1, TUB4/TUB4-mCherry::NAT RPS1/RPS1:: CpCSA6-GFP::URA3*	This study

Supplementary Table 4. Primers used in this study

Name	Sequence (5'-3')	Description
RFP-PstI-F	AAACCCctgcagAAAGATGGTTTCTAAAGGTG	RFP-HygB K7
RFP-NheI-R	CCCAAAgctagcCATATTATTATCTTCAGAAG	RFP-HygB K7
K7_BFP_GFP_Ch4_Right_F	TATATATTTCTGGGCAATGCAGCAATTCTCGGATATCACCGAAAAAAAAGATCTTAGCGGGCACGACACGACTCTCTTGATATAAGCGAATTTTCAGTATCAGGAAACAGCTATGACC	Integration of the RFP-HygB K7 on the right arm of Chr4
RFP_Insertion_Ch4_Right_Reverse	TCTCTATACGAGTTAAGAGTAGTCTTACAATAGTCTATAGATAGAATTTCAGACCTTTTTGTGTGGGTATTGCCGAAATTCTTTTTCCAGAAGATGACGAGAGAAAATACCCGTGACG	Integration of the RFP-HygB K7 on the right arm of Chr4
PJ86	ACAAGCTTATTGAGTGACGAAAAGTC	Confirmation of pNIMX integration
PJ87	TTTACGGGTTGTTAAACCTTCGATTC	
PJ88	ATACTACTGAAAATTTCCTGACTTTC	Confirmation of overexpression plasmid integration
PJ89	ATTACTATTTACAATCAAAGGTGGTC	
PJ90	ATCAACAAGTTTGTACAAA	Sequencing of overexpression plasmid
SR149	CgcACTAGTATGGTTTCAAAAGGTGAAGAAG	Amplification of mCherry-coding gene
SR150	ggaCCCGGGACCCAGAAAGCATTCATCGCG	
LS39FP	TCCCCGCGGGATCGATATAAAACTAATCGTGTTAG	C-term tagging of Tub4 with GFP/mCherry
LS39RP	GGACTAGTTATACCCATATCTGCATCATCTATATTG	
PJ77	tataCCGCGGACTGTTCAATTAGTCGATTGGTGTC	C-term tagging of Tub1 with mCherry
PJ78	atatACTAGTATATTCTTCTTCTTCTTCAGGGAAAG	
NV241	TAA GGG CCC CCA GCT GCT ACT TCC TC	C-term tagging of Cse4 with TAP
NV242	acgc GTCGAC GGCCAATTATAAATGTGAAGGG	
PJ108	atatAGATCTAATAAGAATACGCTATCTCC	C-term tagging of Csa6 with TAP
PJ109	atatTTAATTAAGTTAGAACGACCCATAAATTC	
NV34	GAGCACGTATTGGGTTTGC	Confirmation of TAP cassette integration
PJ127	atatGATATCATGGAAGATTCAACTGAAGATATAATTA	Cloning of *CSA6TAP* under P_{TET}
PJ128	atatGATATCTCACTGATGATTCGCGTC	

PJ110	atatGGTACCAATGCTAGTAGGGTCTAGAC	Deletion cassette for *BUB2*
PJ111	atatCTCGAGTTTGTATCGGAAGGATGTAG	
PJ112	atatCCGCGGATCAATTCTGCACATGGTATG	
PJ113	atatGAGCTCTTGCCTAATAAGACGCCAATTC	
PJ114	atatCCGCGGATATACGCTTTCCCTTCG	
PJ115	atatGAGCTCAATTCTTTAGGAACTTTTCTATCG	
PJ116	AGTCTTGAACGAAAAAGTCTAG	
PJ3	CTATTCTCTAGAAAGTATAGGAACTTC	Confirmation of pPSFS2a integration
PJ118	acgcctaacatatgtgaagtg	
PJ95	atatGGTACCAAGAAGCATGTGGTATGAAGCAC	Deletion cassette for *CSA6*
PJ96	atatCTCGAGTTGTGTCGGTCTGTACGTG	
PJ97	atatCCGCGGGTGGGTAGGTTACACAGAGTC	
PJ98	atatGAGCTCTGGTCCACTACAACCCCTTTTG	
PJ99	TGGCTGATATGGCTCATTG	
PJ93	atgtGGATCCATGGAAGATTCAACTGAAGATATAATTA	Cloning of *CSA6* under *MET3*
PJ94	atctCTGCAGATTATATGCAGCAGATTGAGAAGG	
PJ141	atatGGATCCATAACTCTTTCACGCAAGCTC	C-term tagging of Sol1 with TAP
PJ142	atatTTAATTAATATATTATCAAACGATAATCTCTTTGGTTTG	
PJ119	atatGATATCATGTCCTCTTCTAATGATACACCATC	Cloning of *SOL1* under P_{TET}
PJ120	atatGATATCTTATATATTATCAAACGATAATCTCTTTGGTTTG	
PJ121	atatCCGCGGACAAGAAAGTCTACGCTAAATTC	C-term tagging of Tem1 with GFP
PJ122	atatACTAGTCTTATATATCAATATGGGTTCCCCCAC	
PJ123	TGCTACCATTGGTCTCAAATGATG	
PJ124	ccatacgcgaaagtagtg	Confirmation of GFP cassette integration
PJ147	atatGGATCCACATAACAAATGCCAAACCAAC	C-term tagging of Clb2 with TAP
PJ148	atatTTAATTAACTCTTCTGCTTCTGCTACCAC	
TEJ1	attaCCGCGGAATAAGAATACGCTATCTCC	C-term tagging of Csa6 with mCherry
TEJ2	atgcACTAGTGTTAGAACGACCCATAAATTC	
TEJ13	TCGAAGAAATGCTGTCC	

TEJ14	TCTTCTTCACCTTTTGAAACC	Confirmation of mCherry cassette integration
PJ106	atatCCGCGGAATTGAAGAAAGAGGTTCAAGAC	C-term tagging of Spc110 with GFP
PJ107	atatACTAGTATTGTATTTAAGTCTGGCCAC	
PJ157	atatCCGCGGGTCAACTCTGATGGTTCAATTG	C-term tagging of Cmd1 with GFP
PJ158	atatACTAGTTTTAGCTGCTAATAACAGAGTAAATTC	
PJ159	atatAAGCTTAGAGACGAATGTCAACGATATG	
PJ160	atatGGTACCCTCAAAACCCTTCGGGA	
VS5	AGTCTCTAGACAAGTATTCAACAATTTCTGTC	Ectopic expression of *C. dubliniensis* Csa6, tagged with GFP
VS6	GTGAAAAGTTCTTCTCCCTTACTCATATTGGAATGGCCCATAAATTCTG	
VS7	CAGAATTTATGGGCCATTCCAATATGAGTAAGGGAGSSGAACTTTTCAC	
VS8	AGTCCTGCAGGGGCATTTTATGATGGAATGAATG	
TEJ15	atgcCTCGAGatgagtaagggagaagaacttttc	Cloning of GFP in Cip10
TEJ18	atgcAAGCTTcattttatgatggaatgaatggg	
HA004	ATATGGTACCTACTTGGCATGTTATTGTCGG	Ectopic expression of *C. tropicalis* Csa6, tagged with GFP
HA005	ATATCTCGAGTTTGACTAAGGAAATGCGAAACTGTG	
DB2	cactggtaccgtccgacattgtagtctctaatcag	Ectopic expression of *C. parapsilosis* Csa6, tagged with GFP
DB3	atgcctcgaggttagctggggtcatcaacgtag	

Supplementary Table 5. Plasmids used in this study

Name	Description	Reference
CIp10-P_{TET}-GTW derivatives	Overexpression plasmid collection	[24]
pNIMX	Plasmid harbouring P_{TET} transactivator	[1]
pGFP-HIS	GFP-tagging plasmid	[8]
pRFP-Arg4	mCherry-tagging plasmid	[9]
pFA-TAP-*ARG4*	TAP-tagging plasmid	[16]
pFA-TAP-*HIS1*		
pSFS2a	Recyclable *SAT1*-flipper cassette	[14]
pCaDis	Plasmid for promoter replacement with *MET3*pr	[15]
pBSNAT	Plasmid used for cloning *NAT1*	[11]
pCaADH1-yEmRFP	Plasmid used for cloning mCherry	[10]
pMad2-2	Plasmid used for cloning *CSE4-TAP* fragment	[13]
pNIM1R-RFP	Plasmid used for cloning RFP	[2]
pTDH3-GFP-URA3	Plasmid used for generating the pTDH3-RFP-HygB plasmid	[3]
pAU34-CaHygB	Plasmid used for cloning HygB	[4]
CIp10	Plasmid used for cloning GFP, CtCsa6 and CpCsa6	[18]
pTub4-GFP-His	GFP-tagging plasmid for Tub4	This study
pTub4-mCherry-Arg4	mCherry-tagging plasmids for Tub4	This study
pTub4-mCherry-Nat		
pTub1-mCherry-Arg4	mCherry-tagging plasmid for Tub1	This study
pCse4-TAP-Leu	TAP-tagging plasmid for Cse4	This study
pCsa6-TAP-Arg	TAP-tagging plasmid for Csa6	This study
CIp10-P_{TET}-Csa6TAP	Overexpression plasmid for *CSA6-TAP*	This study
pBub2del#1	Deletion cassettes for *BUB2*	This study
pBub2del#2		
pCsa6del	Deletion cassette for *CSA6*	This study
pCsa6-Met3-Ura	Plasmids for promoter replacement of *CSA6* with *MET3*	This study
pCsa6-Met3-His		
CIp10-P_{TET}-SOL1	Overexpression plasmid for *SOL1*	This study
CIp10-P_{TET}-SOL1TAP	Overexpression plasmid for *SOL1-TAP*	This study
pSol1-TAP-His	TAP-tagging plasmid for Sol1	This study
pTEM1-GFP-His	GFP-tagging plasmid for Tem1	This study
pClb2-TAP-His	TAP-tagging plasmid for Clb2	This study
pCsa6-mCherry-Arg	mCherry-tagging plasmid for Csa6	This study
pSpc110-GFP-His	GFP-tagging plasmid for Spc110	This study
pCmd1-GFP-His #1	GFP-tagging plasmids for Cmd1	This study

pCmd1-GFP-His #2		
pCdCsa6-GFP-ARS2	Ectopic expression of GFP-tagged *C. dubliniensis* Csa6	This study
pCIp10-GFP-CtCsa6	Ectopic expression of GFP-tagged *C. tropicalis* Csa6	This study
pCIp10-GFP-CpCsa6	Ectopic expression of GFP-tagged *C. parapsilosis* Csa6	This study

Supplementary References

1. Chauvel M*, et al.* A versatile overexpression strategy in the pathogenic yeast *Candida albicans*: identification of regulators of morphogenesis and fitness. *PLoS One* **7**, e45912 (2012).

2. Prieto D, Roman E, Correia I, Pla J. The HOG pathway is critical for the colonization of the mouse gastrointestinal tract by *Candida albicans*. *PLoS One* **9**, e87128 (2014).

3. Znaidi S*, et al.* Systematic gene overexpression in *Candida albicans* identifies a regulator of early adaptation to the mammalian gut. *Cell Microbiol.* **20**, e12890 (2018).

4. Basso LR, Jr.*, et al.* Transformation of *Candida albicans* with a synthetic hygromycin B resistance gene. *Yeast* **27**, 1039-1048 (2010).

5. Feri A*, et al.* Analysis of Repair Mechanisms following an Induced Double-Strand Break Uncovers Recessive Deleterious Alleles in the *Candida albicans* Diploid Genome. *mBio* **7**, (2016).

6. Harwood AJ. The rapid boiling method for small-scale preparation of plasmid DNA. *Methods Mol. Biol.* **58**, 265-267 (1996).

7. Walther A, Wendland J. An improved transformation protocol for the human fungal pathogen *Candida albicans*. *Curr. Genet.* **42**, 339-343 (2003).

8. Chatterjee G*, et al.* Repeat-Associated Fission Yeast-Like Regional Centromeres in the Ascomycetous Budding Yeast *Candida tropicalis*. *PLoS Genet.* **12**, e1005839 (2016).

9. Varshney N, Sanyal K. Aurora kinase Ipl1 facilitates bilobed distribution of clustered kinetochores to ensure error-free chromosome segregation in *Candida albicans*. *Mol. Microbiol.* **112**, 569-587 (2019).

10. Keppler-Ross S, Noffz C, Dean N. A new purple fluorescent color marker for genetic studies in *Saccharomyces cerevisiae* and *Candida albicans*. *Genetics* **179**, 705-710 (2008).

11. Thakur J, Sanyal K. Efficient neocentromere formation is suppressed by gene conversion to maintain centromere function at native physical chromosomal loci in *Candida albicans*. *Genome Res.* **23**, 638-652 (2013).

12. Mitra S, Gomez-Raja J, Larriba G, Dubey DD, Sanyal K. Rad51-Rad52 mediated maintenance of centromeric chromatin in *Candida albicans*. *PLoS Genet.* **10**, e1004344 (2014).

13. Thakur J, Sanyal K. The essentiality of the fungus-specific Dam1 complex is correlated with a one-kinetochore-one-microtubule interaction present throughout the cell cycle, independent of the nature of a centromere. *Eukaryot. Cell.* **10**, 1295-1305 (2011).

14. Reuss O, Vik A, Kolter R, Morschhauser J. The SAT1 flipper, an optimized tool for gene disruption in *Candida albicans*. *Gene* **341**, 119-127 (2004).

15. Care RS, Trevethick J, Binley KM, Sudbery PE. The MET3 promoter: a new tool for *Candida albicans* molecular genetics. *Mol. Microbiol.* **34**, 792-798 (1999).

16. Lavoie H, Sellam A, Askew C, Nantel A, Whiteway M. A toolbox for epitope-tagging and genome-wide location analysis in *Candida albicans*. *BMC Genomics* **9**, 578 (2008).

17. Cannon RD, Jenkinson HF, Shepherd MG. Isolation and nucleotide sequence of an autonomously replicating sequence (ARS) element functional in *Candida albicans* and *Saccharomyces cerevisiae*. *Mol. Gen. Genet.* **221**, 210-218 (1990).

18. Murad AM, Lee PR, Broadbent ID, Barelle CJ, Brown AJ. CIp10, an efficient and convenient integrating vector for *Candida albicans*. *Yeast* **16**, 325-327 (2000).

19. Loll-Krippleber R*, et al.* A FACS-optimized screen identifies regulators of genome stability in *Candida albicans*. *Eukaryot. Cell* **14**, 311-322 (2015).

20. Potter SC, Luciani A, Eddy SR, Park Y, Lopez R, Finn RD. HMMER web server: 2018 update. *Nucleic Acids Res* **46**, W200-W204 (2018).

21. Noble SM, Johnson AD. Strains and strategies for large-scale gene deletion studies of the diploid human fungal pathogen *Candida albicans*. *Eukaryot. Cell* **4**, 298-309 (2005).

22. Joglekar AP*, et al.* Molecular architecture of the kinetochore-microtubule attachment site is conserved between point and regional centromeres. *J. Cell Biol.* **181**, 587-594 (2008).

23. Milne SW*, et al.* Role of *Candida albicans* Tem1 in mitotic exit and cytokinesis. *Fungal Genet. Biol.* **69**, 84-95 (2014).

24. Legrand M*, et al.* Erratum: Generating genomic platforms to study *Candida albicans* pathogenesis. *Nucleic Acids Res.* **46**, 8664 (2018).
