# Supplementary material for: A phylogenetically-restricted essential cell cycle progression factor in the human pathogen Candida albicans
Source: Nat Commun. 2022 Jul 23;13:4256. doi: 10.1038/s41467-022-31980-3 (PMC9307598; doi:10.1038/s41467-022-31980-3)
Supplement: Supplementary file 2 — Reporting Summary [file 41467_2022_31980_MOESM2_ESM.pdf]

nature portfolio

Corresponding author(s): Kaustuv Sanyal, Christophe d'Enfert

Last updated by author(s): Jun 30, 2022

Reporting Summary

Nature Portfolio wishes to improve the reproducibility of the work that we publish. This form provides structure for consistency and transparency in reporting. For further information on Nature Portfolio policies, see our Editorial Policies and the Editorial Policy Checklist.

Statistics

For all statistical analyses, confirm that the following items are present in the figure legend, table legend, main text, or Methods section.

n/a	Confirmed	
☐	☒	The exact sample size (*n*) for each experimental group/condition, given as a discrete number and unit of measurement
☐	☒	A statement on whether measurements were taken from distinct samples or whether the same sample was measured repeatedly
☐	☒	The statistical test(s) used AND whether they are one- or two-sided<br>*Only common tests should be described solely by name; describe more complex techniques in the Methods section.*
☒	☐	A description of all covariates tested
☒	☐	A description of any assumptions or corrections, such as tests of normality and adjustment for multiple comparisons
☐	☒	A full description of the statistical parameters including central tendency (e.g. means) or other basic estimates (e.g. regression coefficient) AND variation (e.g. standard deviation) or associated estimates of uncertainty (e.g. confidence intervals)
☐	☒	For null hypothesis testing, the test statistic (e.g. *F*, *t*, *r*) with confidence intervals, effect sizes, degrees of freedom and *P* value noted<br>*Give P values as exact values whenever suitable.*
☒	☐	For Bayesian analysis, information on the choice of priors and Markov chain Monte Carlo settings
☒	☐	For hierarchical and complex designs, identification of the appropriate level for tests and full reporting of outcomes
☒	☐	Estimates of effect sizes (e.g. Cohen's *d*, Pearson's *r*), indicating how they were calculated

Our web collection on statistics for biologists contains articles on many of the points above.

Software and code

Policy information about availability of computer code

Data collection	1. Gels, blots and plate pictures were imaged using ChemiDoc™ Touch (Bio-RAD), Quantity One v.4.6.9, imageLab™ v6.0.0.<br>2. Microscopy images were acquired using Zeiss Zen Blue v2.3.<br>3. Flow cytometry data was acquired using BD FACSDiva™ software version 8.0.1.
Data analysis	1. The pipeline for genome sequence analysis has been published in our previous work PMID: 29884848.<br>2. Homology searches and domain predictions were performed using HmmerWeb v2.41.1 2, Candida Genome database (www.candidagenome.org), Saccharomyces Genome database (www.yeastgenome.org) and NCBI nonredundant protein database (https://blast.ncbi.nlm.nih.gov/Blast.cgi).<br>3. Protein sequences were aligned using Clustal Omega (www.clustal.org) and visualized using Jalview v2.11.1.0.<br>4. Microscopy image processing was done using ImageJ v. 1.51.<br>5. Flow cytometry data was analyzed using FlowJo X 10.0.7r2.<br>6. Image Lab™ v6.0.0 was used to visualize gels, blots and plate pictures.<br>7. Data analysis was performed using GraphPad Prism™v5.00

For manuscripts utilizing custom algorithms or software that are central to the research but not yet described in published literature, software must be made available to editors and reviewers. We strongly encourage code deposition in a community repository (e.g. GitHub). See the Nature Portfolio guidelines for submitting code & software for further information.

Data

Policy information about availability of data

All manuscripts must include a data availability statement. This statement should provide the following information, where applicable:

- Accession codes, unique identifiers, or web links for publicly available datasets
- A description of any restrictions on data availability
- For clinical datasets or third party data, please ensure that the statement adheres to our policy

The source data underlying Fig. 3b; Fig. 5b; Fig. 5f; Fig. 6d; Supplementary Fig. 1e; Supplementary Fig. 1g; Supplementary Fig. 3c-e; Supplementary Fig. 6d; Supplementary Fig. 8e; Supplementary Fig. 9b; and Supplementary Fig. 11 a-b are provided in the Source Data file. Genome sequences have been deposited in the NCBI Sequence Read Archive under BioProject ID PRJNA842202 (https://www.ncbi.nlm.nih.gov/bioproject/842202). Publicly available databases used in the study include Candida Genome database (www.candidagenome.org), Saccharomyces Genome database (www.yeastgenome.org) and NCBI nonredundant protein database (https://blast.ncbi.nlm.nih.gov/Blast.cgi). Strains, plasmids and other data that supports the findings of this study are available upon reasonable request from the corresponding authors.

Field-specific reporting

Please select the one below that is the best fit for your research. If you are not sure, read the appropriate sections before making your selection.

[x] Life sciences [ ] Behavioural & social sciences [ ] Ecological, evolutionary & environmental sciences

For a reference copy of the document with all sections, see nature.com/documents/nr-reporting-summary-flat.pdf

Life sciences study design

All studies must disclose on these points even when the disclosure is negative.

Sample size	No statistical methods were used to predetermine sample size. Sample size and number of replicates were chosen based on other studies with similar methodologies (PMID: 31095812, 33514624, 24973462 and 25595446). In case sample variance was observed, an additional independent experiment was performed to ensure proper interpretation of the results. Where statistical analysis was applied, three independent replicates or n<10 were taken.
Data exclusions	No data were excluded from the analyses
Replication	All the flow cytometric data analyses, spot dilution assays and western blot analyses were performed in either duplicates or triplicates. For all microscopic analyses n≥30 to n≥100 was taken. All the experiments were reliably reproduced, information related to independent experiments were specified in the figure legends or methods.
Randomization	Strains and conditions that were directly compared were typically cultured together. Microscopy image acquisition was performed randomly. All samples were allotted randomly into experimental groups. Further randomization was not applicable.
Blinding	Blinding was technically difficult as knowledge about the treatments and conditions was required for the researchers to be able to standardize, perform or analyze the experiments.

Reporting for specific materials, systems and methods

We require information from authors about some types of materials, experimental systems and methods used in many studies. Here, indicate whether each material, system or method listed is relevant to your study. If you are not sure if a list item applies to your research, read the appropriate section before selecting a response.

Materials & experimental systems

n/a	Involved in the study	
[ ]	[x]	Antibodies
[x]	[ ]	Eukaryotic cell lines
[x]	[ ]	Palaeontology and archaeology
[x]	[ ]	Animals and other organisms
[x]	[ ]	Human research participants
[x]	[ ]	Clinical data
[x]	[ ]	Dual use research of concern

Methods

n/a	Involved in the study	
[x]	[ ]	ChIP-seq
[ ]	[x]	Flow cytometry
[x]	[ ]	MRI-based neuroimaging

Antibodies

Antibodies used	1. Rabbit anti-protein A antibody (no. P3775; Sigma, 1:5000)<br>2. Mouse anti-PSTAIRE antibody (no. ab10345; Abcam, 1:5000)<br>3. Goat Anti-Mouse IgG H&L (HRP) (no. ab97023; Abcam, 1:10000)<br>4. Goat Anti-Rabbit IgG H&L (HRP) (no. ab97051; Abcam, 1:10000)
Validation	Rabbit anti-protein A antibody (no. P3775; Sigma) and Mouse anti-PSTAIRE antibody (no. ab10345; Abcam) have been used previously in our previous work PMID: 22536162. The specificity of Goat Anti-Mouse IgG H&L (HRP) (no. ab97023; Abcam) and Goat Anti-Rabbit IgG H&L (HRP) (no. ab97051; Abcam) in immunoblotting was determined by Abcam.

Flow Cytometry

Plots

Confirm that:

- [x] The axis labels state the marker and fluorochrome used (e.g. CD4-FITC).
- [x] The axis scales are clearly visible. Include numbers along axes only for bottom left plot of group (a 'group' is an analysis of identical markers).
- [x] All plots are contour plots with outliers or pseudocolor plots.
- [x] A numerical value for number of cells or percentage (with statistics) is provided.

Methodology

Sample preparation	For BFP/GFP analysis, appropriate dilutions of live cells in 1XPBS were used. For cell cycle analysis, we fixed cells using ice-cold 70% ethanol overnight followed by RNAse treatment and propidium iodide staining. Detailed protocols have been mentioned in the methods section.
Instrument	FACSAria III, BD Biosciences, The MoFlo® Astrios™
Software	FlowJo X 10.0.7r2
Cell population abundance	The purity of the BFP-GFP+ cells after sorting was determine by both marker analysis and flow cytometry. For marker analysis, we replica plated BFP-GFP+ colonies along with the appropriate control strains on CM-Arg, CM-His and YPDU+hyg B (800 µg/ml). The colonies from CM-Arg plates were further analyzed for BFP, GFP and RFP markers by flow cytometry. For BFP/GFP LOH analysis we acquired at least a million events. For cell cycle analysis, we acquired at least 30, 000 events.
Gating strategy	For CSA reporter system, an untagged genetic background (SN148) was used to define the boundaries between BFP/GFP/RFP channels. For propidium iodide staining, an RNAse-treated unstained sample was used as a negative control.

- [x] Tick this box to confirm that a figure exemplifying the gating strategy is provided in the Supplementary Information.
