# Supplementary material for: A phylogenetically-restricted essential cell cycle progression factor in the human pathogen Candida albicans
Source: Nat Commun. 2022 Jul 23;13:4256. doi: 10.1038/s41467-022-31980-3 (PMC9307598; doi:10.1038/s41467-022-31980-3)
Supplement: Supplementary file 3 — Description of Additional Supplementary Files [file 41467_2022_31980_MOESM3_ESM.pdf]

Title: Supplementary Data 1

Description: Results of the primary screen involving overexpression of 1067 genes
